# Unique Dental Morphology of *Homo floresiensis* and Its Evolutionary Implications

**DOI:** 10.1371/journal.pone.0141614

**Published:** 2015-11-18

**Authors:** Yousuke Kaifu, Reiko T. Kono, Thomas Sutikna, Emanuel Wahyu Saptomo, Jatmiko, Rokus Due Awe

**Affiliations:** 1 Department of Anthropology, National Museum of Nature and Science, Tokyo, Japan; 2 Department of Biological Sciences, The University of Tokyo, Tokyo, Japan; 3 Centre for Archaeological Science, University of Wollongong, Wollongong, Australia; 4 The National Research and Development Centre for Archaeology, Jakarta, Indonesia; University of Hawaii at Manoa, UNITED STATES

## Abstract

*Homo floresiensis* is an extinct, diminutive hominin species discovered in the Late Pleistocene deposits of Liang Bua cave, Flores, eastern Indonesia. The nature and evolutionary origins of *H*. *floresiensis*’ unique physical characters have been intensively debated. Based on extensive comparisons using linear metric analyses, crown contour analyses, and other trait-by-trait morphological comparisons, we report here that the dental remains from multiple individuals indicate that *H*. *floresiensis* had primitive canine-premolar and advanced molar morphologies, a combination of dental traits unknown in any other hominin species. The primitive aspects are comparable to *H*. *erectus* from the Early Pleistocene, whereas some of the molar morphologies are more progressive even compared to those of modern humans. This evidence contradicts the earlier claim of an entirely modern human-like dental morphology of *H*. *floresiensis*, while at the same time does not support the hypothesis that *H*. *floresiensis* originated from a much older *H*. *habilis* or *Australopithecus*-like small-brained hominin species currently unknown in the Asian fossil record. These results are however consistent with the alternative hypothesis that *H*. *floresiensis* derived from an earlier Asian *Homo erectus* population and experienced substantial body and brain size dwarfism in an isolated insular setting. The dentition of *H*. *floresiensis* is not a simple, scaled-down version of earlier hominins.

## Introduction

Previous studies showed that *Homo floresiensis* exhibits unusually small body and brain sizes for a Late Pleistocene *Homo* [1], a *H*. *erectus*-like cranial shape [2,3], an *Australopithecus*-like upper vs. lower limb proportion [4–7], and other primitive, advanced, and unique skeletal features [8–14]. Researchers agree that this unique mosaic has significant evolutionary meaning, but disagree on what it is. Some anticipate that *H*. *floresiensis* evolved from an *Australopithecus* or *H*. *habilis*-like, primitive, small-brained hominin currently unknown in the Asian fossil record and thus signals unexpectedly early hominin dispersal into this region [14–19], whereas others suggest that the species is an example of substantial body and brain size insular dwarfism from Asian *H*. *erectus* [1,2,20,21]. In this paper, we present the first comprehensive analyses of their dental morphology to further contribute this debate.

Teeth are one of the most informative elements in hominin evolutionary studies [22], and this is no exception in the case of *H*. *floresiensis*. Unlike postcranial elements, abundant comparative fossil specimens are available for the teeth. Indeed, only one cranium of *H*. *floresiensis* currently exists (LB1), but dental remains represent up to three individuals for each tooth. Nevertheless, interpretations of the *H*. *floresiensis* dental morphology has been controversial, with opinion divided if their teeth are primitive [15] or modern like *H*. *sapiens* [23]. “How primitive” is another unresolved question. For example, there is a claim that the mesiodistally elongated P_3_ of *H*. *floresiensis* represents a very primitive hominin condition not seen in *H*. *erectus* [15], but more detailed analyses of this and other dental traits are needed to assess its taxonomic affinity [24].

**Fig 1:**
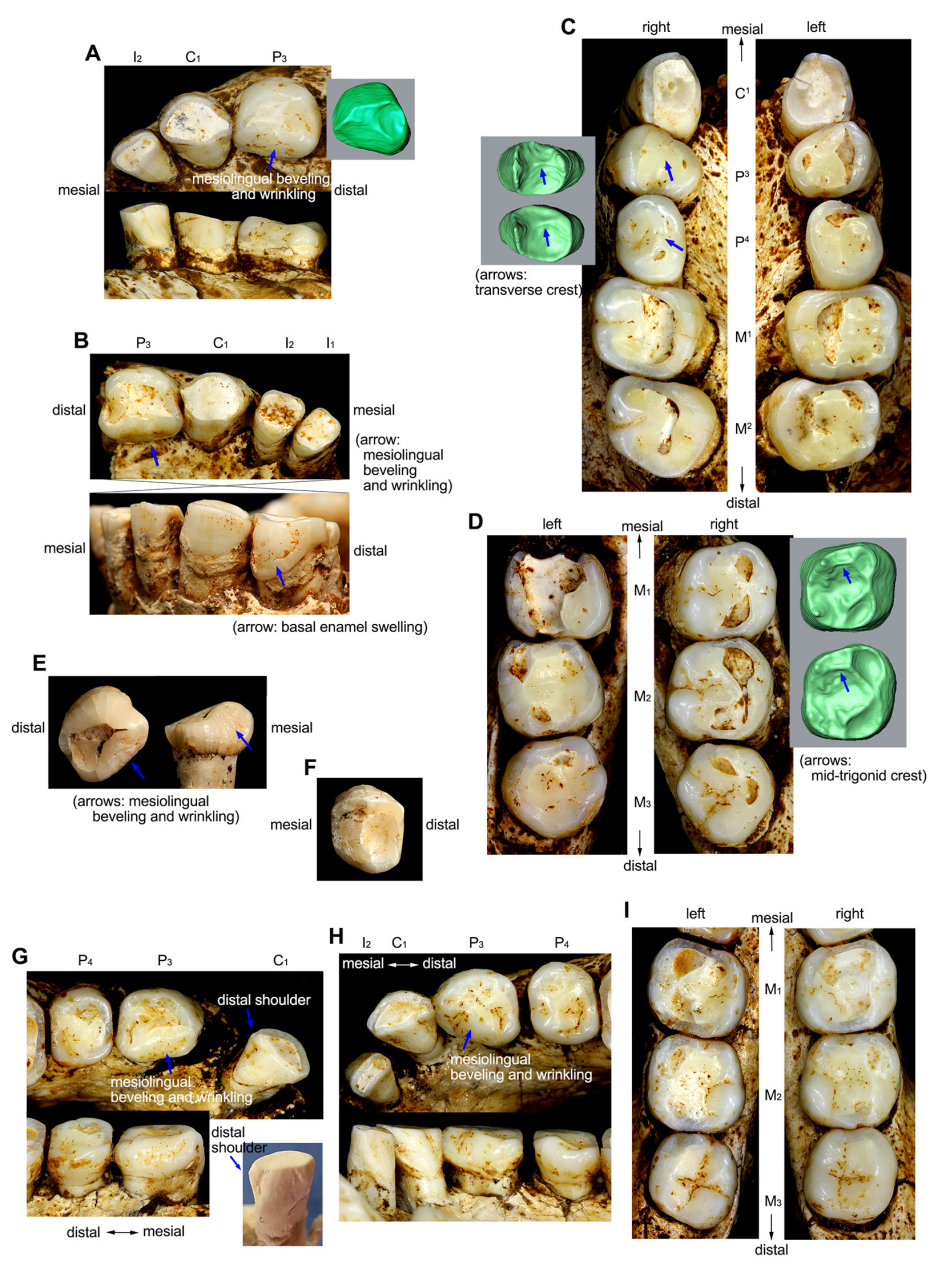
Teeth of *Homo floresiensis*. Right (A) and left (B) anterior dentitions of the LB1 mandible. (C) Maxillary dentition of LB1 with EDJ surface images for the right P^3^ and P^4^. (D) Mandibular molars of LB1. (E) Occlusal (left) and lingual (right) views of the LB2/2 left P_3_. (F) Occlusal view of the LB15/1 right P_4_. Left (G) and right (H) anterior dentitions of the LB6/1 mandible with a photograph of a cast of its left C_1_ (with blue background). (I) Mandibular molars of LB6/1. See ref. [24] for LB 15/2 (I^1^) and LB6/14 (I_1_).

The currently available *H*. *floresiensis* dental sample is comprised of one nearly complete maxillary dentition (LB1), two almost complete mandibular dentitions (LB1, LB6/1), and four isolated teeth (I^1^ [LB15/2], I_1_ [LB6/14], P_3_ [LB2/2], P_4_ [LB15/1]) (Fig 1). All of these specimens are housed at the National Research and Development Centre for Archaeology, Jakarta. Full morphological descriptions of these specimens are available elsewhere along with a reassessment of the previous studies [24]. The purpose of the present study is to examine general dental evolutionary trends in Early Pleistocene *Homo* with particular emphasis on *H*. *floresiensis*’ two major ancestral candidates, the earliest *Homo* from Africa (*H*. *habilis sensu lato*: >2.3―1.6 Ma [million years ago]) and early Javanese *H*. *erectus* (>1.2―0.8 Ma) [2], and evaluate *H*. *floresiensis* within this framework. Comparisons are also made with a sample of global modern humans including local prehistoric populations (N = 490) to examine dental morphological modernity in *H*. *floresiensis*. Three analytical approaches are employed: 1) traditional metric analyses based on crown length and breadth data, 2) comparisons of crown contour using normalized Elliptic Fourier Analysis (EFA), and 3) non-metric and linear metric comparisons of individual morphological traits not captured by the above two analyses.

## Materials and Methods

### Comparative samples

Comparative fossil *Homo* samples are from the Early Pleistocene of Africa (*H*. *habilis s*. *l*., *H*. *ergaster*), Caucasia (Dmanisi *Homo*), and Indonesia (early Javanese *H*. *erectus*) as listed in S1 Table. These include the claimed two major ancestral candidates for *H*. *floresiensis* (*H*. *habilis* and early Javanese *H*. *erectus*). We focused on these Early Pleistocene samples to investigate the evolution of *H*. *floresiensis* because of its generally primitive cranial and other skeletal morphology [2,10,11,13,14,17], with the expectation that larger-brained hominins could not be the ancestor of this small-brained species [25], as well as the evidence for the presence of hominins (stone artifacts) on Flores ~1.0 Ma [26]. For linear metric and non-metric analyses, data from East Asian Middle Pleistocene archaic *Homo* specimens were also included. All necessary permits were obtained for the described study, which complied with all relevant regulations. Studies on the original specimens from Kenya (Turkana) and Tanzania (Olduvai) were conducted under the research permits No. NCST 5/002/R/124 issued by the National Council of Science and Technology, Kenya, and No. 2008-324-NA-2008-124 issued by the Tanzania Commission for Science and Technology.

Debate continues if *H*. *habilis s*. *l*. includes diverse evolving lineages [27–30], but we pooled the relevant specimens from East Africa for the present purpose to recognize the primitive morphological condition in earliest *Homo*. The early Javanese *H*. *erectus* sample can be further divided into two chronological subsamples: ‘Sangiran Lower’ (those from the upper Sangiran and the lowest Bapang Formations dated to ≥1.2−1.0 Ma) and ‘Sangiran Upper’ (those from the middle-lower part of the Bapang Formation dated to 1.0−0.8 Ma) (chronology based on [31], but see [32]). The postcanine tooth crowns are distinctly reduced from the former to the latter [33–35]; Previous studies demonstrated that the Sangiran Lower sample is derived compared to *H*. *habilis* in cranial, mandibular, and dental morphology, and can be called a primitive form of *H*. *erectus* [33,36–38]. Our ‘East Asian Middle Pleistocene archaic *Homo*’ sample is different from traditional Middle Pleistocene Chinese *H*. *erectus* (e.g., [39]); We pooled a diverse taxa including *H*. *erectus* and post-*erectus* grade *Homo* for the present comparison as a broad reference sample for archaic dental morphology of the post-Early Pleistocene period in eastern Asia.

Our *H*. *sapiens* sample is from Africa, Europe, Asia, and Oceania, with particular emphasis on prehistoric individuals from Southeast Asia including Flores as well as modern small-bodied populations (Philippine Negrito, Andaman, African Pygmy, Bushman) (S2 Table). This choice was made to reflect species-wide variation of *H*. *sapiens*, and to respond to the claim that Liang Bua *H*. *floresiensis* resembles a local short-statured Australomelanesian population [23]. Sexes were pooled due to general difficulties in sex assignment for variously fragmentary hominin fossils.

### Materials

We analyzed morphology of the permanent teeth. Specimens with severe wear were excluded from the metric analyses. Metric and non-metric data were obtained from the original specimens, excellent-quality plaster casts, or literature (S1 Table). For all the *H*. *floresiensis*, early Javanese *H*. *erectus*, and *H*. *sapiens* specimens, “isolated” plaster casts were prepared by Y.K with partial assistance from Hisao Baba (S1 Fig). Silicone was used for molding the original specimens and the resulting plaster cast was then cut with a saw to isolate individual teeth. Such isolated casts can be measured more easily and accurately than when the original specimens are still housed in the jaw bones making collecting accurate linear measurement data more difficult. Thus, we used these isolated casts for linear measurement and crown contour extraction. Non-isolated, high-quality plaster casts were used for most of the *H*. *habilis* and *H*. *ergaster* specimens. These were prepared by Gen Suwa with reported dimensional accuracies within ±0.1 mm [40].

### Measurement

We used a digital sliding caliper (Mitutoyo Co.) to measure mesiodistal (MD) and buccolingual (BL) crown diameters with allowance for wear, following the methods described in ref. [41]. Values from the right and left sides were averaged for the fossil specimens, while the data for *H*. *sapiens* (casts from the 490 individuals) were available only from the better-preserved and/or less-worn side due to limitation of the time available for molding. The MD and BL measurements for a small number of African and Georgian fossil *Homo* and most of the Zhoukoudian as well as Xujiayao specimens were taken from the literature [41–46]. The remaining metric data were collected directly by Y.K.

### Size-adjusted PCAs

We performed principal component analyses (PCAs) based on size-adjusted MD and BL crown diameters. Because LB1 lacks the M^3^ and P_4_, the maxillary analysis included P^3^, P^4^, M^1^, and M^2^, and the mandibular analysis P_3_, M_1_, M_2_, and M_3_. The size-adjustment was done by dividing each of the MD and BL diameters by ‘crown size factor’, which was calculated as square root of the average crown area for each individual (average of the ‘MD x BL’ for all the four teeth included for each PCA). Group means for *H*. *floresiensis* (LB1, LB6/1) and nine chrono-regional subsamples of *H*. *sapiens* (prehistoric Southeast Asian, Philippine Negrito, New Guinea, Australia/Tasmania Aborigine, modern Indonesian, Bushman, African Pygmy, East African, and German) were used to compute variance-covariance matrices. PC scores for a small number of the available Early Pleistocene *Homo* individuals were also calculated using the equations derived from the samples of *H*. *sapiens* and *H*. *floresiensis*.

### Elliptic Fourier Analysis (EFA)

Occlusal crown contours of maxillary molars (M^1^ and M^2^) and mandibular premolars and molars (P_3_, P_4_, M_1_, and M_2_) were analyzed by normalized (size-standardized) EFA (elliptic Fourier analysis) [47,48], a method that does not require homologous landmarks and thus is suitable for moderately worn tooth crowns. Two or more *H*. *floresiensis* individuals are available for the four mandibular teeth. The right P^3^ and P^4^ of LB1 are relatively unworn but they were excluded from this analysis. The mesiobuccal corner of the P^3^, one of the most diagnostic point for this tooth in *Homo* [49], has been lost by the contact with the C^1^; The orientation of P^4^ is difficult due to the damage on its distal cervical line (See Fig 1C in [24]). The M^2^s of LB1 are markedly asymmetrical [24]. In consideration of this observation, both sides were included for *H*. *floresiensis* if the wear is not very severe (M^1^ and M^2^ of LB1, and P_3_, M_1_, and M_2_ of LB6). The contours of the comparative specimens were taken from the better-preserved side. Comparisons are made on the images from the right teeth or horizontally flipped images of the left teeth. The crown contour of each tooth is captured by digital photography with a dental cast placed so that its cervical line is perpendicular to the axis of the camera lens [50–52]. Fluctuations of the cervical lines are ignored [40]. For example, the buccal cervical lines of the *H*. *floresiensis* P_3_s deviate considerably toward their root apices (Fig 1B). The orientations of these teeth are defined without referring to this buccal part of the line.

A special system was used to minimize errors associated with photography (parallax effect, orientation of the tooth and scale, etc.), as addressed in S2 Fig. Images were uploaded into Canvas X software (ACD Systems) and backgrounds were removed using a semi-automated process. In worn teeth the original occlusal crown contour was reconstructed on the digital image with reference to each dental cast (S3 Fig). The thick dental calculus deposits on the right M^1^, M_1_ and M_2_ of LB1 were cleaned virtually on the micro-CT imagery (S3A and S3B Fig). Before calculating normalized Elliptical Fourier descriptors (EFDs), each tooth crown was aligned along its MD axis. Capturing of crown contours from the digital images, obtaining EFDs, and PCA of the normalized EFDs were conducted using the software SHAPE 1.3 [53]. Between-group differences in the PC scores were tested by Mann-Whitney U Test with and without Bonferroni correction. The Bonferroni correction is a method used to avoid problems with Type I errors in multiple comparisons. In this method, the statistical significance level is adjusted simply by being divided by the number of hypotheses being tested. This correction becomes very conservative when the number of comparisons is large and the tests are not independent [54]. This is probably the case for the present PCAs, because there may be some correlations between the outlines of different teeth (e.g., among the premolars or molars), and the number of the hypotheses is as many as 24.

### Non-metric and linear metric comparisons

In order to examine dental traits that are potentially useful to distinguish earlier and later *Homo* but were not captured by the above EFAs, we made another set of comparative analyses based on observed trait frequency (presence/absence) and linear metric data. The traits observed are listed in S3 Table. These were selected with reference to previous studies [33,40,43,45,49,50,52,55–69] and our own preliminary observations, and were restricted by the preservation of the *H*. *floresiensis* materials. Fisher’s exact test, a method usually used when dealing with sample sizes, was employed for two-sample comparisons in frequency data.

## Results

### Crown size

**Fig 2:**
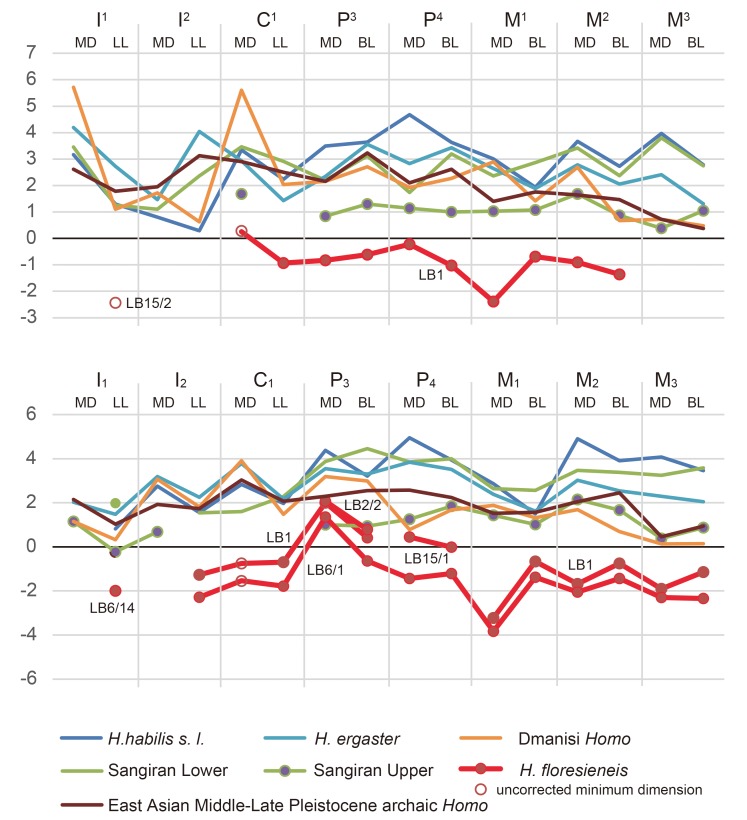
Z-scores for the tooth crown length (MD) and breadth (LL or BL) for *H*. *floresiensis* and other fossil *Homo* groups as compared to the global *H*. *sapiens* sample. Z-scores are relative deviations from the *H*. *sapiens* means in units of standard deviation. Note that the Dmanisi *Homo* sample here is based on the two smaller individuals. Due to sever tooth wear the largest individual (D4500/2600) l was excluded [27].

We first analyzed tooth size based on crown length (MD) and breadth (LL or BL) data (Fig 2). Many of the *H*. *floresiensis* teeth are within the smaller range of variation exhibited by the global *H*. *sapiens* sample. Remarkable deviations from this general trend are disproportionately long P_3_s as well as short M^1^ and M_1_ in *H*. *floresiensis*.

**Fig 3:**
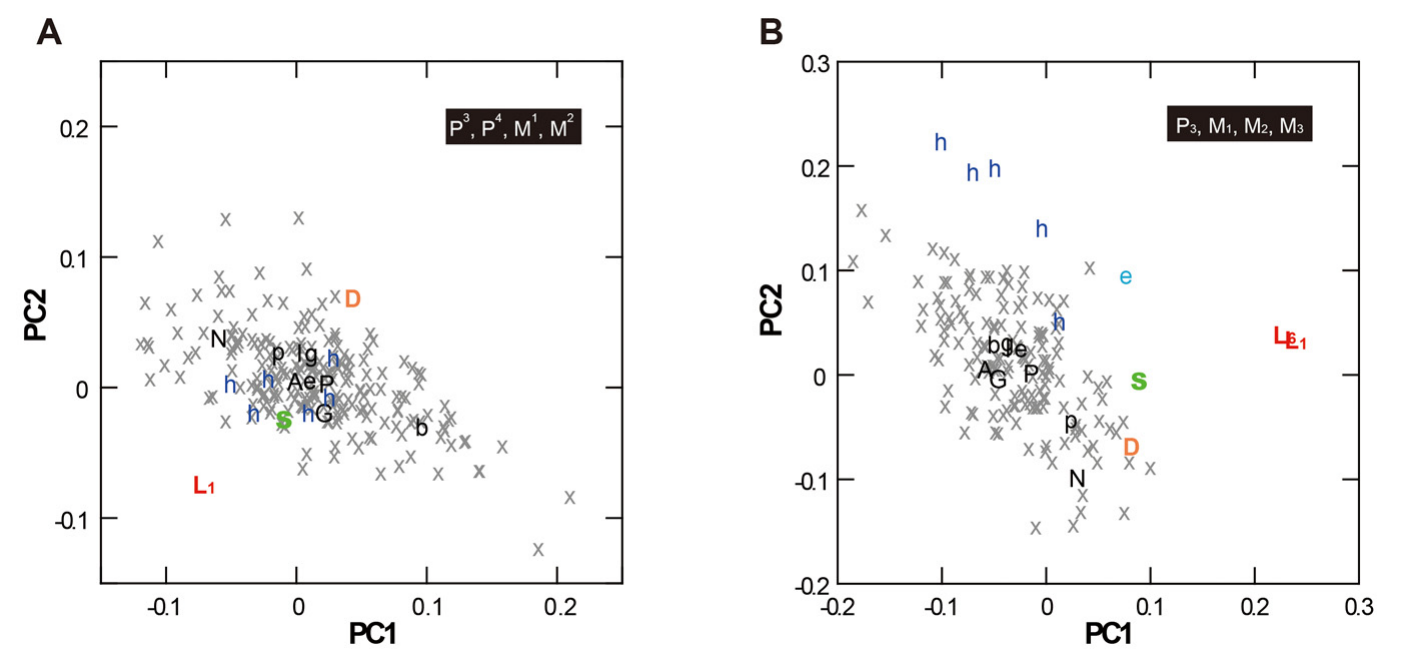
Results of the PCAs based on size-adjusted MD and BL crown diameters. Analyses of the maxillary (A) and mandibular (B) teeth. Black letters = *H*. *sapiens* subsample means (P = prehistoric Southeast Asia, N = Philippine Negrito, G = New Guinea, A = Australia/Tasmania Aborigine, I = modern Indonesian, b = Bushman, p = African Pygmy, e = East African, g = German); Gray crosses = *H*. *sapiens* individuals; Colored letters = archaic *Homo* individuals (L_1_ = LB1, L_6_ = LB6/1, S = early Javanese *H*. *erectus* [Sangiran 4, 22], D = Dmanisi [D2282/211, 2700/2735], e = *H*. *ergaster* [KNM-ER 992], h = *H*. *habilis* [L894-1; Omo75-14G; KNM-ER 1590, 1802, 1813, 60000; OH 13, 16, 39]).

This unique tooth size proportion was also confirmed by the following multivariate analyses. Fig 3 and Table 1 are the results of principal component analyses based on size-adjusted MD and BL data. The generated PC1 and PC2 cumulatively explain 75% (maxilla) or 91% (mandible) of the total variation. These PCs show no (PC1 and PC2 for the maxillary analysis, and PC1 for the mandibular analysis) or only slight (PC2 for the mandibular analysis) correlations with the ‘crown size factor’ (Table 1), and thus reflect crown shape variations that are mostly independent from crown size. In both analyses, the PC scores of the *H*. *floresiensis* teeth occupy unique positions relative to the *H*. *sapiens* individuals.

**Table 1:** Component loadings and other results of the group-mean PCA based on size-adjusted crown diameters.

	Maxilla		Mandible	
Variables	PC1	PC2	PC1	PC2
P1 MD	-0.58	-0.05	0.97	0.18
P1 BL	-0.94	0.07	0.89	-0.38
P2 MD	-0.76	-0.46		
P2 BL	-0.74	0.61		
M1 MD	0.76	0.53	-0.93	-0.29
M1 BL	-0.22	-0.63	0.70	-0.44
M2 MD	0.67	-0.65	-0.64	-0.22
M2 BL	0.31	0.43	0.75	-0.16
M3 MD			-0.80	0.59
M3 BL			-0.34	0.19
Proportion of the variance (%)	49	26	79	12
Cumulative proportion (%)	49	75	79	91
Pearson’s *r* with the ‘crown size factor’^a^	-0.05	-0.06	-0.07	0.26

^a^Correlation coefficient based on the sample of the *H*. *sapiens* individuals.

The Early Pleistocene *Homo* individuals, which were projected onto the PC spaces using the above variance-covariance matrices, occupy a space between *H*. *floresiensis* and *H*. *sapiens* in the analysis of the mandibular teeth, suggesting that the pattern observed in *H*. *floresiensis* involves primitive morphology for the genus *Homo*. Such a trend is, however, not evident in the analysis of the maxillary teeth. In both analyses, the early Javanese *H*. *erectus* individuals (Sangiran 4 for the maxillary teeth and Sangiran 22 for the mandibular teeth) are comparatively close to the positions of *H*. *floresiensis*.

### Crown contour

**Fig 4:**
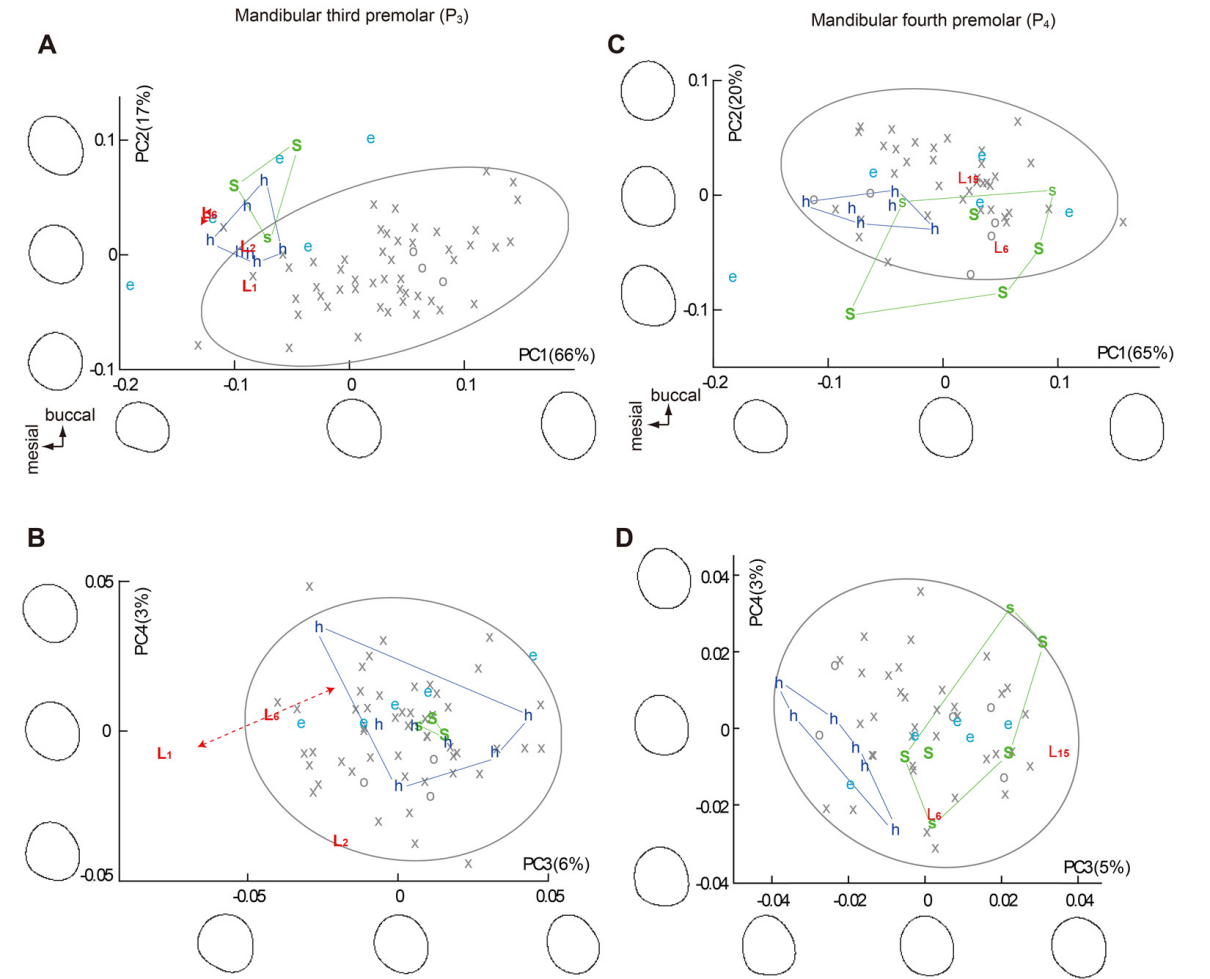
Plots of PC scores derived from the normalized Elliptic Fourier Analyses (EFAs) on the crown contours (mandibular premolars). (A) and (B) Mandibular first premolar. (C) and (D) Mandibular second premolar. Symbol and color codes: gray symbols = *H*. *sapiens* (crosses = Southeast Asia/Melanesia/Australia, circles = African Pygmy); colored letters = fossil *Homo* (L_1_ = LB1, L_2_ = LB2/2, L_6_ = LB6/1, L_15_ = LB15/1, S = early Javanese *H*. *erectus* (Sangiran Lower), s = early Javanese *H*. *erectus* (Sangiran Upper), D = Dmanisi, e = *H*. *ergaster*, h = *H*. *habilis*). The right and left teeth are included for *H*. *floresiensis* when available and they are indicated by the dashed line with arrow heads. The crown outlines for -2 SD, 0, and +2 SD, 95% confidence ellipses for the *H*. *sapiens* sample, and ranges for *H*. *erectus* and *H*. *habilis* samples are shown. Proportion of the variance explained by each PC is in the parentheses.

**Fig 5:**
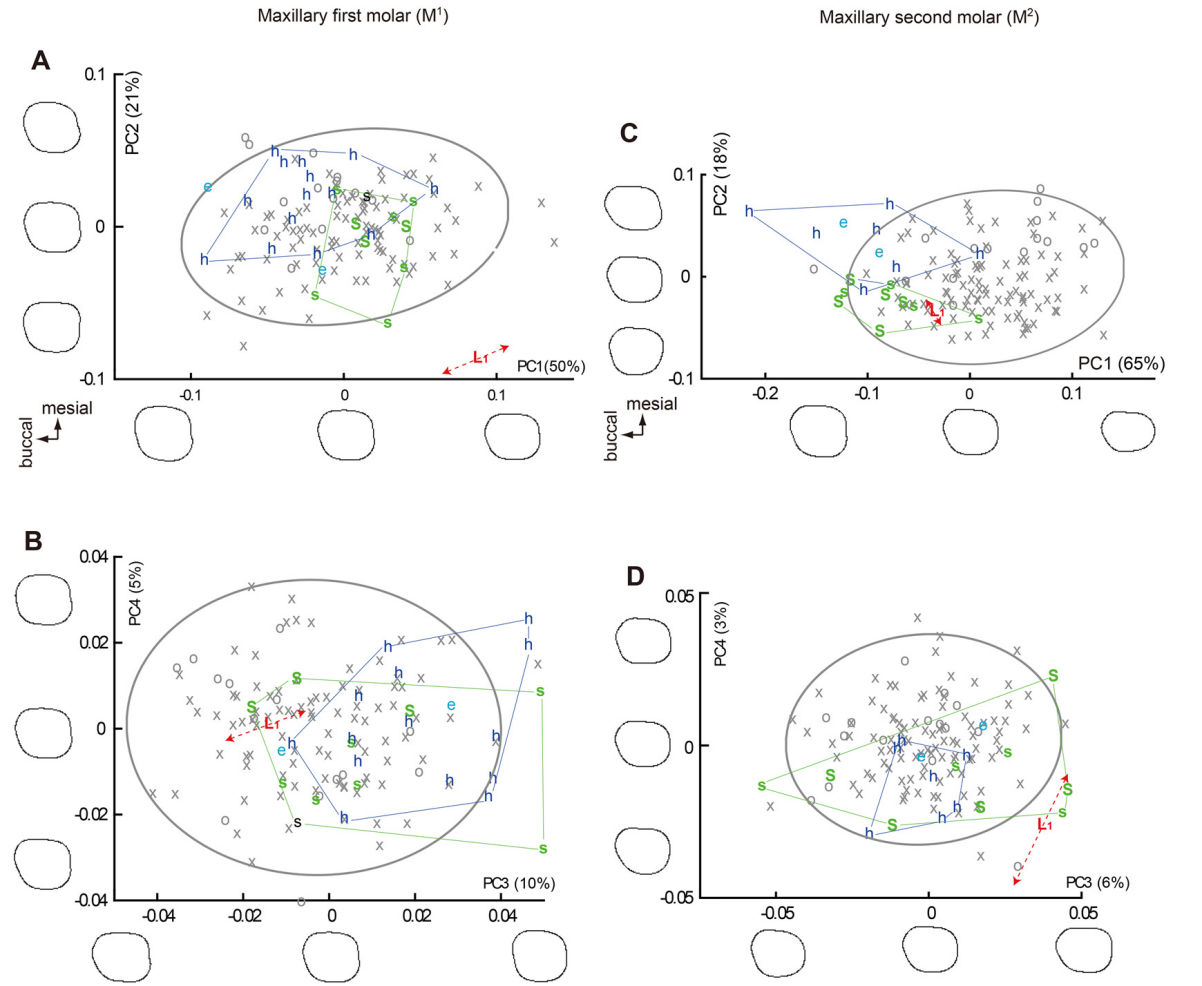
Plots of PC scores derived from the normalized Elliptic Fourier Analyses (EFAs) on the crown contours (maxillary molars). (A) and (B) Maxillary first molar. (C) and (D) Maxillary second molar. See Fig 4 for notes.

**Fig 6:**
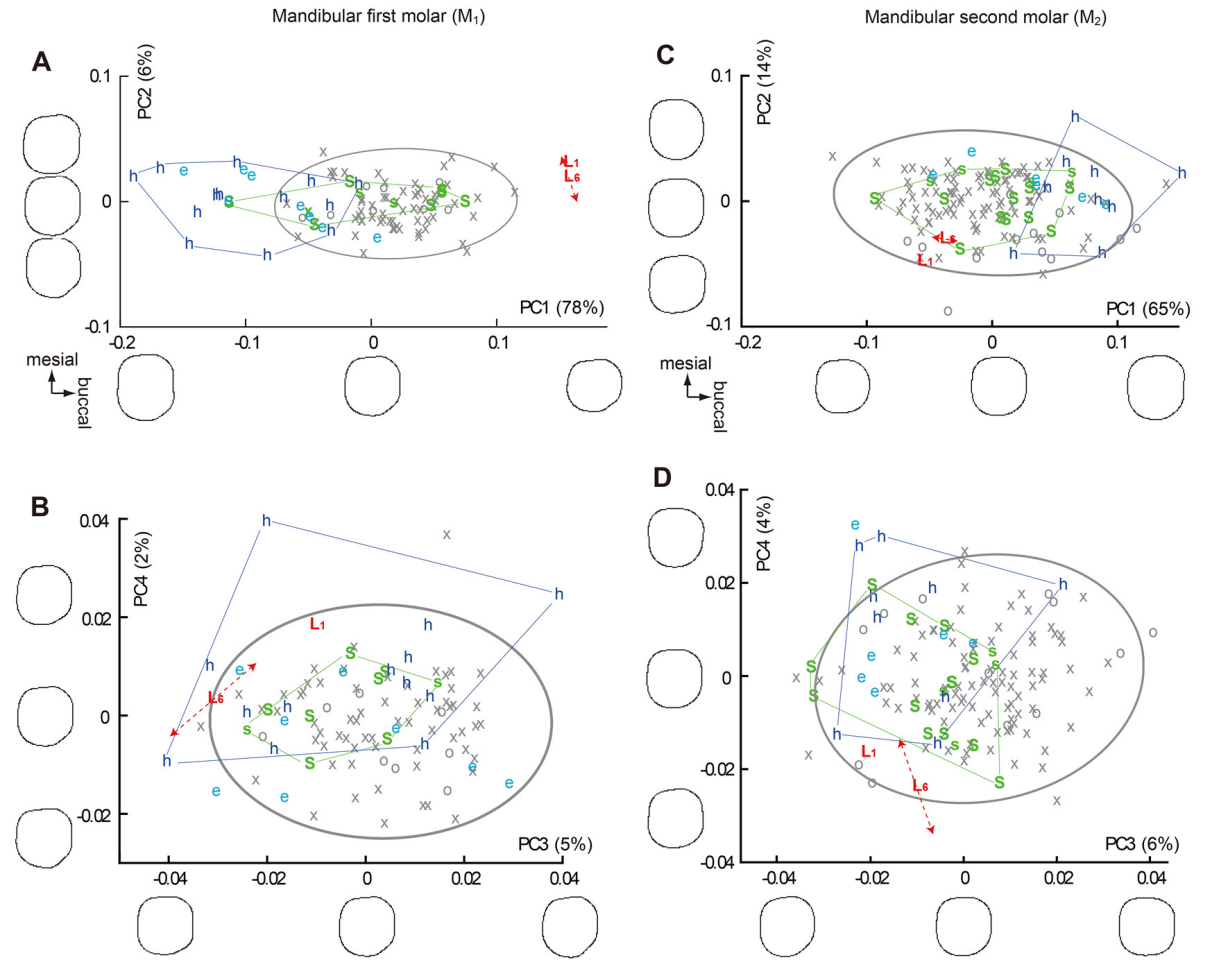
Plots of PC scores derived from the normalized Elliptic Fourier Analyses (EFAs) on the crown contours (mandibular molars). (A) and (B) Mandibular first molar. (C) and (D) Mandibular second molar. See Fig 4 for notes.

Because wear obscures much of the occlusal surface morphology of the *H*. *floresiensis* teeth, we analyzed the occlusal crown contours of six teeth (P_3_, P_4_, M^1^, M^2^, M_1_, and M_2_) by normalized (size-adjusted) Elliptic Fourier Analysis (EFA) combined with PCA. In consideration of the previous claim that the Liang Bua Pleistocene hominins resemble a local short-statured Australo-Melanesian population [23], the *H*. *sapiens* sample used here includes prehistoric people with Australo-Melanesian affinities sampled from Flores, Java, Malaysia, and Vietnam [70], as well as Aboriginal Australians, Papuans, Philippine Negritos, and African Pygmies (N = 54 [P_3_], 41 [P_4_], 106 [M^1^], 112 [M^2^], 71 [M_1_], 105 [M_2_]: S2 Table). Fossil *Homo* samples from Africa (*H*. *habilis* and *H*. *ergaster*) and Java (early *H*. *erectus*) were included (S1 Table). Within the *H*. *sapiens* sample, each PC1 shows a weak correlation with measured crown area (Pearson’s correlation coefficients were -0.17 [P_3_], 0.22 [P_4_], -0.13 [M^1^], -0.26 [M^2^], 0.15 [M_1_], and 0.11 [M_2_]; M^2^ was the only tooth that reached statistical significance at the α level of 0.05), indicating that the size-adjustment was effective for these analyses. In all six analyses the first two PCs (PC1 and PC2) cumulatively explain 71−85%, and the next two (PC3 and PC4) 7−15% of the total variations (see Figs 4, 5 and 6 for the value for each PC). These four PCs are considered below.

**Fig 7:**
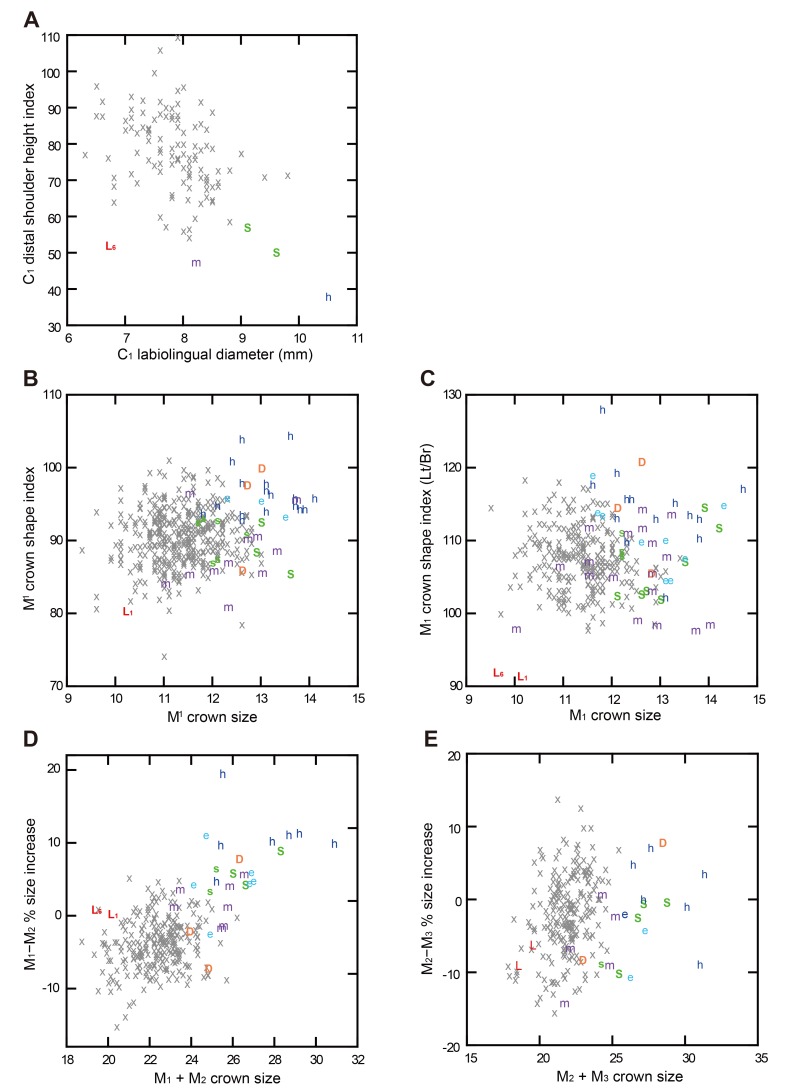
Relationships between tooth crown size and selected morphological traits. (A) C_1_ crown shape. (B) and (C) M_1_ crown shape. (D) and (E) Mandibular molar size proportions. The ‘crown size’ is square root of the computed crown area (MD × BL diameters).

Because the two chronological samples of early Javanese *H*. *erectus* (Sangiran Lower and Sangiran Upper) are essentially similar to each other in all the PCs, they were pooled for the following statistical analyses. In the PC scores plotted in Figs 4, 5 and 6, the pooled Early Pleistocene fossil *Homo* sample (*H*. *habilis*, *H*. *ergaster*, and early Javanese *H*. *erectus*) differ significantly from *H*. *sapiens* in eight out of the twenty-four PCs generated from the six EFAs (PC1 and PC2 for P_3_, PC3 for M^1^, PC1 and PC4 for M^2^, PC1 and PC3 for M_2_: *P* < 0.05, Mann-Whitney U Test with Bonferroni correction). In particular, PC1 and PC2 for P_3_ in combination separate the modern and the Early Pleistocene samples nearly completely. Two other PCs also differ significantly if Bonferroni correction is not made (PC2 for P_4_, PC3 for M_1_). Thus, these eight (with Bonferroni correction) or ten (without Bonferroni correction) PCs reflect primitive features for the genus *Homo*. Figs 4, 5 and 6 shows that *H*. *floresiensis* shares all of these primitive features except for PC1 of M_1_. In PC 1 of M_1_ as well as PC1 and PC2 for M^1^, *H*. *floresiensis* is distinct from both modern and fossil *Homo* (Figs 5A and 6A). These primarily reflect the short MD diameters of the first molars (Fig 2). In the *H*. *sapiens* sample, there is no evidence that smaller first molars approach the short configuration similar to *H*. *floresiensis* (Fig 7B and 7C). This further highlights the uniqueness of the latter.

**Fig 8:**
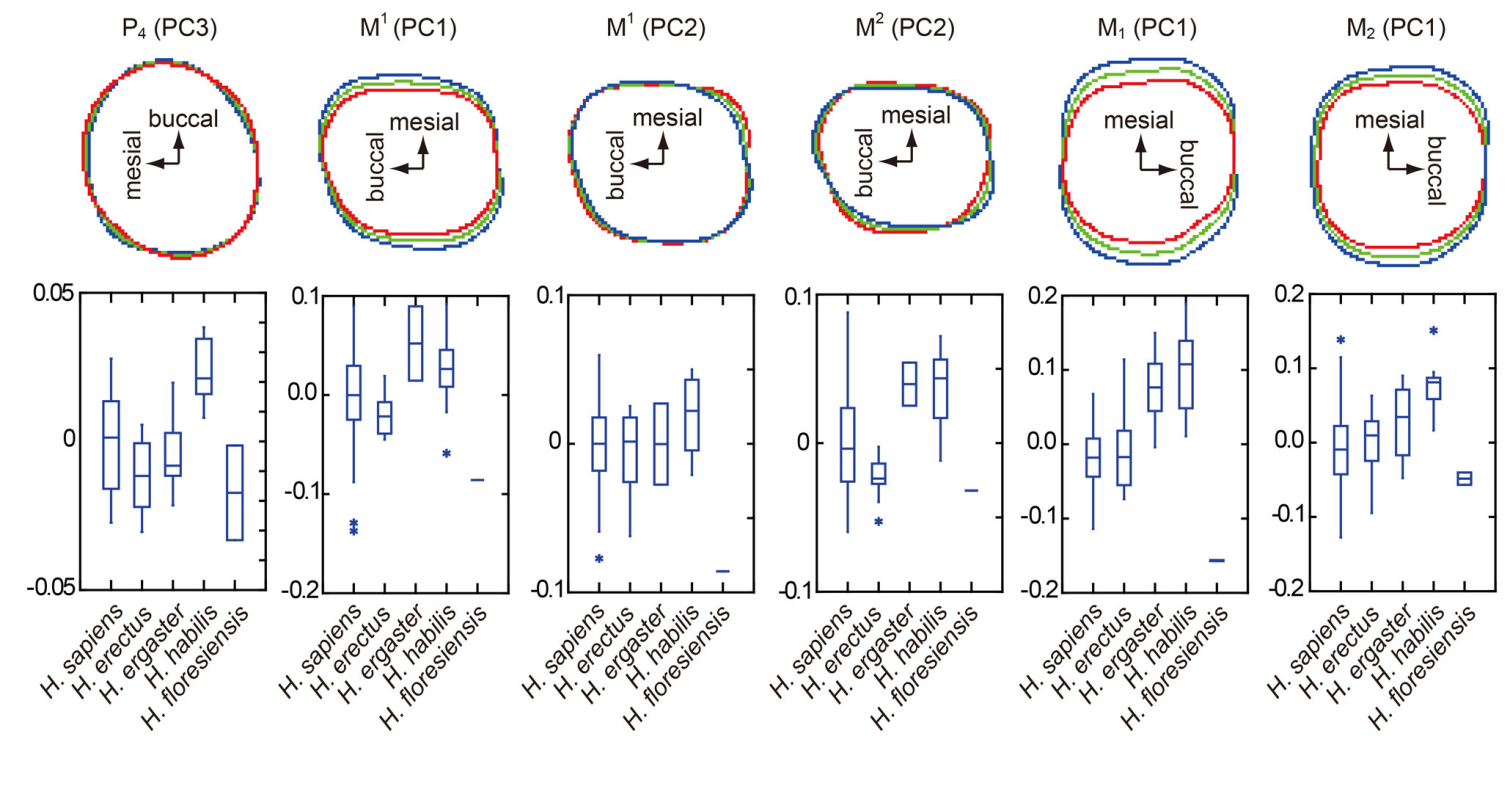
Six PCs showing significant differences in crown contours between *H*. *habilis* and early Javanese *H*. *erectus*. The positive and negative are reversed from Figs 4, 5 and 6 in PC3 for P_4_, PC1 for M^1^, and PC1 for M_1_, for the sake of unanimity in the directions of variation. See Figs 4, 5 and 6 for component loading for each PC. The shape variation reflected by each PC is shown in the upper row. Blue and red lines indicate contours of +2 SD and −2 SD of the entire sample, respectively. The green line is the grand mean. In all of these six cases, the contours of *H*. *floresiensis* are close to the red outlines, and those of *H*. *habilis* to blue outlines. Box plots of the PC scores are shown in the lower row. Note that *H*. *habilis* plots far from *H*. *floresiensis* whereas early Javanese *H*. *erectus* is closer to *H*. *floresiensis* in all these PCs.

**Fig 9:**
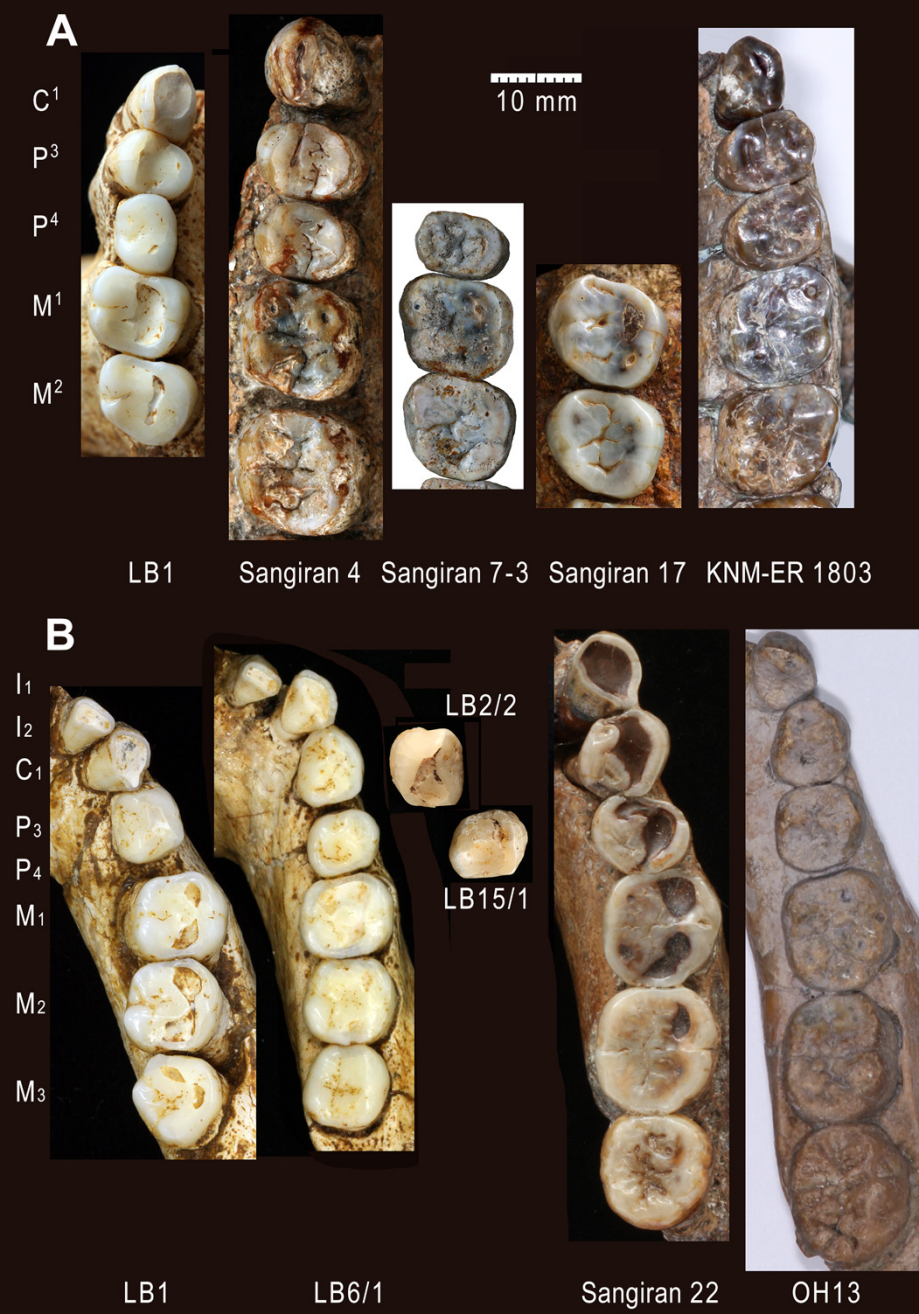
Dentitions of *H*. *floresiensis* and selected Early Pleistocene *Homo* specimens. Maxillary (A) and mandibular (B) dentitions of *H*. *floresiensis* (LB), early Javanese *H*. *erectus* (Sangiran), and *H*. *habilis* (KNM-ER and OH).

When the PC score for the early Javanese *H*. *erectus* and the *H*. *habilis* samples are compared to each other, the former differs significantly from the latter in having MD short and BL wide M_1_ (PC1) and M_2_ (PC1) [62,71], and a BL symmetric M^2^ (PC2) [72] (*P* < 0.05, Mann-Whitney U Test with Bonferroni correction). A non-triangular P_4_ (PC3) [52], and a MD short (PC1) and BL symmetric (PC2) M^1^ are also added to the above list if Bonferroni corrections are not made. In all of these six distinguishing characters (Fig 8), the Liang Bua Pleistocene teeth are similar (P_4_, M^2^ and M_2_) or closer (M^1^ and M_1_) to early Javanese *H*. *erectus* but are remote from *H*. *habilis* (Fig 9). Thus, the postcanine crown contours of *H*. *floresiensis* are derived relative to *H*. *habilis* and more similar to early Javanese *H*. *erectus* in many aspects.

### Other morphological traits

Twenty morphological characters of individual teeth (nos. 1−20) were assessed based on presence/absence with metric criteria when applicable, and three characters of the dentition (nos. 21−23) were evaluated based on linear metric data. Among these 23 traits listed in S3 Table, 16 were found to be of some use to evaluate dental morphological status of *H*. *floresiensis*. These 16 characters are reported in Table 2 and described below. The rest of the results are available in S3 Table and S1 Text. Differences in the frequency data were tested between the *H*. *habilis* and other samples, and between the *H*. *sapiens* and other samples (Table 2). Because the results showed no differences between the crown contours of the older and younger early Javanese *H*. *erectus* specimens, they were pooled for the statistical tests in Table 2.

**Table 2:** Results of the non-metric and linear metric comparisons.

		*H*. *habilis*	*H*. *erg*.	Dmanisi	eJ *H*. *erectus* (Lower, Upper)^c^	MP East Asia	*H*. *sapiens*	*H*. *floresiensis* (status)^d^
**Non-metric comparisons of individual teeth** (frequency and ratio)^a^								
1	C_1_ distal shoulder low	1/1	−	−	2/2**	1/1	*3/109	1/1 (EP-MP)
	(vs. high)						3%	
2	P^3^ transverse crest present	5/11**	1/5	0/1	3/9** (2/5, 1/4)	1/8	**6/283	1/1 (EP)
		45%	20%		33%	13%	2%	
3	P^4^ transverse crest present	3/10**	2/3**	1/2*	4/8** (1/4, 3/4)	1/8	**5/279	1/1 (EP)
		30%			50%	13%	2%	
4	P^3^ buccal groove(s) present	12/12**	**1/5	0/1	6/9 (4/5, 2/4)	**2/6	**139/245	0/1 (post-Hh)
		100%	20%		67%	33%	57%	
6	P^4^ lingual crown MD extensive	8/8**	1/2	2/2*	4/4** (2/2, 2/2)	*2/6	**33/198	1/1 (EP)
		100%			100%	33%	17%	
7	P_3_ lingual cusp posi. mesially	9/9	*3/6	1/2	3/3 (2/2, 1/1)	8/9	163/197	0/3 (post-Hh)
	(vs. distally)	100%	50%			89%	83%	
9	P_3_ mesiolingual crown	0/9	0/6	0/2	0/4 (0/3, 0/1)	0/9	0/214	3/3 (unique)
	beveled and wrinkled	0%	0%		0%	0%	0%	
11	P_4_ transverse crest present	0/7	*5/7**	0/2	*4/6* (3/4, 1/2)	**5/6**	40/220	2/2 (post-Hh)
		0%	71%		67%	83%	18%	
14	P_3_ buccal basal enamel	1/6*	0/5	1/3?*	1/3* (1/3, −)	1/7*	*0/207	1/3 (EP-MP)
	thickened (cingulum)	17%	0%			14%	0%	
15	P_3_ root bifurcated	4/13**	2/5*	1/1	3/6** (3/6, −)	0/7	**26/599^e^	2/3 (EP)
	(vs. fused or single)	31%	40%		50%	0%	4%	
17	M_1_ four-cusped	0/13	0/10	0/2	0/9 (0/7, 0/2)	0/14	9/268	2/2 (Hs)
	(vs. five-cusped)	0%	0%		0%	0%	3%	
18	M_2_ four-cusped	0/9**	0/8**	0/2	0/15** (0/12, 0/3^f^)	0/12**	**163/279	2/2 (Hs)
	(vs. five-cusped)	0%	0%		0%	0%	58%	
19	M_1_ mid-trigonid crest present	0/7	1/9	2/2	4/9** (3/6, 1/3)	1/9	7/176	1/1
		0%	11%		44%	11%	4%	
**Metric comparisons of dentition as a whole** (minimum / maximum, and sample size in the lower rows)^b^								
21	Relative P_3_ size (%)	24 / 26	26 / 28	26 / 27	(26, −)	26 / 28	22 / 27	30 (unique)
		N = 5	N = 3	N = 2	N = 1, −	N = 3	N = 188	N = 2
22	Molar size % increase: M_1_→M_2_	+5 / +20	−2 / +11	−2 / +8	(+4 / +9, +4 / +7)	−2 / +4	−15 / +5	0 / +1 (post-Hh)
		N = 7	N = 6	N = 3	N = 3, 2	N = 6	N = 250	N = 2
23	Alv. arcade index (Lth/Bth, %)	117 / 156	98	123	(107 / 108, −)	88	−	103 / 105 (eJHe)
		N = 5	N = 1	N = 2	N = 2, −	N = 1	−	N = 2

^a^Observed frequencies and percent ratios (for those samples with N ≥ 4) as well as the results of the Fisher’s exact tests for the non-metric comparisons (nos. 1−20). The asterisk(s) to the right of each frequency indicates significant difference from the *H*. *sapiens* sample, and that on the left significant differences from the *H*. *habilis* sample (*: *P* < 0.05, **: *P* < 0.01).

^b^The minimum and maximum values, and sample sizes (in the lower rows) are shown for the metric comparisons (nos. 21−23).

^c^Frequencies for the Sangiran Lower and Upper subsamples in the parentheses.

^d^Morphological status of *H*. *floresiensis* in the parentheses: ‘EP’, a primitive condition shared with the Afro-Asian Early Pleistocene *Homo*; ‘EP-MP’, a primitive condition shared with the Afro-Asian Early Pleistocene *Homo* and the East Asian Middle-Late Pleistocene archaic *Homo*; ‘post-Hh’, a condition derived from *H*. *habilis*; ‘Hs’, a derived condition shared with *H*. *sapiens*; ‘eJHe’, a condition most similar to early Javanese *H*. *erectus*; ‘unique’, a unique condition restricted to *H*. *floresiensis*.

^e^Sample studied by Shields [73].

^f^Zanolli [35] recently suggested the presence of 4-cusped M_2_s in this group.

#### C_1_ distal shoulder height (no. 1 in Table 2)

Canines of *H*. *sapiens* often exhibit an elevated distal shoulder that gives an incisor-like appearance to its crown. We metrically examined this character using the following index: distal shoulder height / labiolingual (LL) crown diameter. The height is the minimum distance between the distal cervical line and the distoincisal corner of the crown.

The resulting index values were: 38% (OH 7: *H*. *habilis*); 50% (Sangiran 22) and 57% (Sangiran 7–58) (early Javanese *H*. *erectus*); 48% (*Sinanthropus* 70: Chinese *H*. *erectus*); and the modern human specimens range between 54% and 110% with the mean value being 78.4% (N = 109). When we categorized each specimen as having a ‘low’ or ‘high’ distal shoulder with the cut-off point of the index value set at 57.5, all three Early Pleistocene *Homo* C_1_s (as well as the Middle Pleistocene Chinese *H*. *erectus* C_1_) were categorized as ‘low’ whereas only three out of the 109 *H*. *sapiens* specimens exhibit this primitive condition (Table 2). The frequency for each of the archaic *Homo* samples was significantly different from that in *H*. *sapiens*, suggesting that a *H*. *sapiens*-like spatulate C_1_ appeared or became dominant after *H*. *erectus/ergaster*. Other East African Early Pleistocene C_1_s (OH 13, OH 16, KNM-ER 992) apparently had similarly low distal shoulder morphology although metric data are not available for these worn or damaged specimens.

The single *H*. *floresiensis* C_1_ available for this metric comparison (LB6/1: Fig 1G), whose distal shoulder remains unworn, also shares this primitive morphology (index = 52). The distal C_1_ shoulder height for another *H*. *floresiensis* individual (LB1) cannot be measured due to wear, but the rounded mesial aspect and the convergent distal aspect of the occlusal outline of its exposed dentine (Fig 1A and 1B) suggests that the same also applies to this individual. In the *H*. *sapiens* sample, there is a weak correlation between the crown size (square root of the computed crown area) and the distal shoulder height index (r = −0.408, or −0.400 if logarithmic transformations are made) so that a smaller C_1_ tends to have a relatively higher distal shoulder. The *H*. *floresiensis* C_1_s retain the low distal shoulder morphology despite being smaller than the modern human average in its LL diameter (Fig 7A).

#### P^3^ and P^4^ transverse crest (nos. 2 and 3 in Table 2)

A transverse crest on a maxillary premolar was recorded when the longitudinal groove does not continue as a deep, continuous groove at the bottom of the occlusal surface but is more or less interrupted by a crest formed by occlusal ridges of the buccal and lingual cusps (corresponding to the category 2 of ref. [59]), regardless of its position (i.e., mesial, middle, or distal on the longitudinal groove). Such a crest formation is rare in our global *H*. *sapiens* sample (2% for both P^3^ [N = 283] and P^4^ [N = 279]) as well as in an European-dominated *H*. *sapiens* sample studied by Martinón-Torres et al. [65] (N = 124−132), but relatively common in the Early Pleistocene *Homo* (35% [P^3^] and 43% [P^4^] for our pooled Early Pleistocene sample) with significant differences between many pairs between the *H*. *sapiens* and the Early Pleistocene samples (Table 2). The P^3^ and P^4^ of *H*. *floresiensis* (LB1: Fig 1C) both exhibit this primitive trait (both on the enamel and EDJ surfaces) that is shared with *H*. *habilis* and early Javanese *H*. *erectus*.

#### P^3^ buccal grooves (no. 4 in Table 2)

Premolars of *Australopithecus* and earlier *Homo* variably express vertical grooves on the mesial and/or distal aspects of their buccal faces [40,55,58]. Here, we compared the frequencies of appearance of one or both of these buccal grooves as opposed to their total absence. Only distinct grooves were counted; specimens with faint grooves/furrows or occlusally situated short grooves were recorded as absent to include slightly worn teeth.

Buccal groove(s) are present in all the *H*. *habilis* P^3^s examined (N = 12), but 30‒40% or more of the P^3^s lack these grooves in later *Homo* groups, with the observed frequencies significantly different in many pair-wise comparisons between the *H*. *habilis* and the later *Homo* samples. In our sample, groove-free P^3^s appear only after *H*. *habilis*. Thus, the total absence of P^3^ buccal grooves in *H*. *floresiensis* (LB1: Fig 1C) is a derived condition at least relative to *H*. *habilis*.

#### P^4^ lingual crown development (no. 6 in Table 2)

The previous geometric morphometric analyses [49] demonstrated that the most marked change in the crown shapes of the *Homo* maxillary premolars is reduction of the lingual crown. *Australopithecus afarensis*, *Au*. *africanus*, and *H*. *habilis* often exhibits a primitive configuration of MD pronounced lingual cusp that is equal to or slightly exceeds the buccal cusp, whereas a lingually tapering crown shape is a typical observation in more recent *Homo* [55,74–76]. Although we did not include the LB1 maxillary premolars in our EFA, the above known trend can be examined in part for P^4^ by a simple comparison between the buccal and lingual crown MD diameters.

In the present study, a P^4^ was recorded as ‘lingual crown MD extensive’ in Table 2 when its maximum MD dimensions were buccal ≤ lingual. A few *H*. *sapiens* specimens develop a large distal accessory tubercle that obscures the above crown configuration. These specimens were recorded as missing data. The result (Table 2) shows that the *H*. *habilis*, the Dmanisi *Homo*, and the early Javanese *H*. *erectus* samples include a significantly higher frequency of specimens with a well-developed lingual crown than in *H*. *sapiens*, consistent with the previous findings [49]. Interestingly, a considerable number (4/6) of the East Asian Middle-Late Pleistocene archaic *Homo* P^4^s exhibit the advanced, reduced lingual crown morphology (Zhoukoudian, Chaoxian, and Xujiayao). The *H*. *floresiensis* P^4^ (LB1: Fig 1C) shows a primitive ‘buccal = lingual’ configuration but is different from a more primitive ‘buccal < lingual’ configuration often observed in *H*. *habilis* (5/8) and Dmanisi *Homo* (2/2).

#### P_3_ lingual cusp position (no. 7 in Table 2)

This trait was assessed with reference to the protoconid apex and the axis of the mesial and distal protoconid crests [40]. The lingual cusp (metaconid) position was recorded as ‘mesial’ when it is located slightly mesial or opposite to the buccal cusp (protoconid). All nine P_3_s of *H*. *habilis* display this primitive pattern reported for *Au*. *afarensis* [40,61,77] and is associated with a MD spacious talonid, but the lingual cusp is located distally relative to the buccal cusp in a few post-*habilis Homo* specimens (Table 2). This latter pattern is found in Dmanisi *Homo* (D211 [right only], 2735 [right only]) and African *H*. *ergaster* (KNM-ER 992, KNM-WT 15000 [left only]; OH 22), as well as Zhoukoudian *H*. *erectus* (Zdansky P_3_), suggesting that it emerged after ~1.75 Ma in *Homo* [24]. A distally located P_3_ lingual cusp is also occasionally found in *H*. *sapiens* (28/197 = 14%), although its differences from the *H*. *habilis* sample is not significant probably because of the relative rareness of this morphology. The three *H*. *floresiensis* P_3_s (LB1, LB2/2, LB6/1) exhibit this derived, distally oriented lingual cusp placement [24] (Fig 1A, 1B, 1E, 1G and 1H).

#### P_3_ mesiolingual beveling (no. 9 in Table 2)

A hominin P_3_ is usually associated with a moderately thick and high mesial marginal ridge (that is often continuous but is occasionally interrupted by a vertical groove) as well as a distinct anterior fovea (either in the form of a pit or a slit). The *H*. *floresiensis* P_3_s are unique showing poor development of both of these structures. Instead, their entire mesiolingual occlusal surface is flattened and finely wrinkled, and is beveled mesially and lingually [24] (Fig 1A, 1B, 1E, 1G and 1H). We failed to find a single specimen with comparable morphology in our P_3_ samples of *H*. *habilis* (N = 9), *H*. *ergaster* (N = 6), Dmanisi *Homo* (N = 2), early Javanese *H*. *erectus* (N = 4), Chinese archaic *Homo* (N = 9), as well as *H*. *sapiens* (N = 214).

#### P_4_ transverse crest (no. 11 in Table 2)

Transverse crest on mandibular premolars were recorded based on the same criterion used for the maxillary premolars (nos. 3 and 4). The available small sample suggests that a P_4_ transverse crest that intervenes in the longitudinal groove appeared in post-*habilis* grade *Homo* (Table 2). *Homo ergaster*, early Javanese *H*. *erectus*, and the Middle Pleistocene East Asian archaic *Homo* display a significantly higher frequency of occurrence of this crest than in *H*. *habilis*. However, the observed frequencies significantly decrease from these post-*habilis* archaic *Homo* groups to the *H*. *sapiens*. Thus frequent occurrence of P_4_ transverse crest is probably a primitive feature shared among post-*habilis* archaic *Homo*. This crest is commonly observed also in the European Middle-Late Pleistocene archaic *Homo* (~86%) [65,66,68]. At least one *H*. *floresiensis* individual (LB6/1) and probably a second (LB15/1) show this condition that is derived compared to *H*. *habilis* (Fig 1F–1H).

#### P_3_ buccal basal enamel swelling (cingulum) (no. 14 in Table 2)

Buccal enamel of canine, premolar, and molar teeth of *Australopithecus* occasionally show cingulum-like basal swelling to form a distinct band along the cervical line [55,74,76,78,79]. We counted this primitive morphology in our *Homo* samples focusing on P_3_. Such a structure was found in OH 6, Sangiran 22, and *Sinanthropus* 70 (a specimen belonging to the BI mandibles). P_3_ of D2735 also has a similar structure (Martinón-Torres, personal communication) although it was previously described as a feature associated with enamel hypoplasia [43]. None of the *H*. *sapiens* P_3_s we examined exhibit basal enamel swelling on their buccal faces (N = 207), but one of the three *H*. *floresiensis* P_3_s (LB1) clearly possesses this primitive character (Fig 1B).

#### P_3_ root form (no. 15 in Table 2)

Wood et al. [60] schematized variation and evolution of mandibular premolar root number and spatial arrangement in *Paranthropus* and *Homo*. Although *Australopithecus* lacks a clear evolutionary trend in premolar root configuration [80], *Homo* shows a general trend of root number reduction from two-rooted patterns (‘MB + D’ pattern for P^3^ and ‘M + D’ pattern for P^4^, respectively) to a single-root form through varying forms of fusion between the two root components [60]. In the present study, we compiled frequency data of bifurcated versus fused/single mandibular premolar roots from the literature [33,40,41,43,45,62,73,81,82]. When distinct mesial and distal root components are fused to each other along their buccal margins [80], the specimens were counted as two-rooted.

The results in Table 2 show that this trait is polymorphic in most samples compared here [60], but the Early Pleistocene *Homo* samples show bifurcated roots significantly more than in the large global modern human sample studied by Shield [73]. Two of the three *H*. *floresiensis* individuals exhibit bifurcated P_3_ roots that are arranged in ‘MB + D’ pattern (LB1, 6/1) and the other individual has a fused (Tomes’) root (LB2/2) [24]. Given the rareness of the two-rooted P_3_s in *H*. *sapiens* (4%, N = 599) [73], the comparatively high frequency of this morphology in *H*. *floresiensis* (2/3) probably reflects its primitiveness [15]. More detailed morphometric analyses are needed to further investigate this issue (e.g., refs. [83–85]).

#### M_1_ cusp number (no. 17 in Table 2)

The absence of a hypoconulid on the M_1_ (four-cusped M_1_) is a trait unknown in *H*. *habilis*, *H*. *ergaster*, Dmanisi *Homo*, early Javanese *H*. *erectus* [43], as well as the Middle Pleistocene archaic hominin dental collection from China [45,81,86], although there is a report that two mid-Middle Pleistocene M_1_s from Atapuerca-Sima de los Huesos, Spain, exhibit this morphology [65]. A four-cusped M_1_ is also rare in modern humans [68], with an observed frequency of 3% in our global sample (N = 269) and 1% in a much larger sample (N = 6790: Appendix A in ref. [87]). Although this frequency is as high as 10% in modern Europeans and Northern Africans, the reported frequencies are much lower in modern human populations from the other regions of the world. Despite its rarity, the M_1_s from the two *H*. *floresiensis* individuals (LB1, 6/1) lack the hypoconulid and are four-cusped [24] (Fig 1D and 1I). In our modern human sample, 4-cusped M_1_s are significantly smaller than 5-cusped M_1_s (*P* = 0.01, two-tailed t-test). This relationship raises a possibility that the loss of the M_1_ hypoconulid occurred independently in *H*. *floresiensis* associated with its unusual crown shortening (Figs 2 and 6A).

#### M_2_ cusp number (no. 18 in Table 2)

All of the fossil M_2_s examined in this study (the archaic *Homo* samples from the Early Pleistocene of Africa, Caucasus, and Java, as well as the Middle Pleistocene of East Asia) have five major cusps, although possible occurrence of four-cusped M_2_s in early Javanese *H*. *erectus* has recently been reported [35,88]. Four-cusped M_2_s are also reported for several individuals of the European Middle Pleistocene archaic *Homo* (Atapuerca-Sima de los Huesos) [65]. This condition is quite common in *H*. *sapiens* [63,68,87] (59% in Table 2).

The M_2_s from two *H*. *floresiensis* individuals are four-cusped [24] (LB1, 6/1: Fig 1D and 1I). As in the case for the M_1_, the loss of a hypoconulid occurred probably in association with the crown size reduction [68]. Four-cusped M_2_s are significantly smaller than 5-cusped M_2_s in our *H*. *sapiens* sample (*P* = 0.000, two-tailed t-test). Scott and Turner [87] observed a similar association in their sample of Pima Indians.

#### M_1_ mid-trigonid crest (no. 19 in Table 2)

A mid-trigonid crest (terminology follows ref. [69]) that bridges between the protoconid and metaconid and borders the distal aspect of the anterior fovea is common in the M_1_s and M_2_s of Neanderthals and European Middle Pleistocene *Homo* but relatively rare in *H*. *sapiens* [65–67,89]. Martinón-Torres et al. [43,64] suggested that this crest characterizes Eurasian archaic hominins as compared to African *H*. *habilis* and *H*. *ergaster*(see also ref. [90]), although other researchers caution that this crest takes variable forms [67] and more detailed studies are needed than a simple presence/absence dichotomy.

Still, our data indicate this simple method can be used to distinguish *Homo* taxa [43,64]. In our samples, this crest occurs significantly more often on the M_1_s of early Javanese *H*. *erectus* than in *H*. *sapiens* (*P* = 0.003, Fisher’s exact test). A distinct mid-trigonid crest is absent in the available seven *H*. *habilis* M_1_s, but present in the two Dmanisi M_1_s [43]. Although the presence/absence of a mid-trigonid crest is obscured in the LB1 M_1_ by wear, the strong expression of a crest on its EDJ surface (Fig 1C; Ref. [24]) strongly suggests that the crest was originally present on its enamel surface [67,69]. Therefore, this *H*. *floresiensis* individual probably shares the crested M_1_ with the Eurasian Early Pleistocene *Homo* groups.

#### P_3_ relative size (no. 21 in Table 2)

As demonstrated in the main text and Fig 2, *H*. *floresiensis* is unique in having a remarkably large P_3_. In this section, we further examine this trait by comparing the relative MD lengths of P_3_/[P_3_+M_1_+M_2_]) [24]. The data in Table 2 show that none of the archaic *Homo* (N = 14) and *H*. *sapiens*(N = 188) specimens reach the high index values exhibited by the two *H*. *floresiensis* individuals, confirming the remarkable relative P_3_ size in this species. Allometry does not explain this unique morphology of *H*. *floresiensis* because its tooth size is within the variation of *H*. *sapiens* (Fig 2), and the smaller-toothed *H*. *sapiens* tend to show smaller relative P_3_ MD lengths than in the larger-toothed archaic *Homo* specimens (Table 2).

#### Molar size proportion (no. 22 in Table 2)

During the course of *Homo* evolution, the posterior molars experienced more marked size reduction than in the first molar, resulting in an alteration of the molar size sequence from plesiomorphic ‘M1 < M2 ≥ M3’ to derived ‘M1 > M2 > M3’ [56,91]. In the present comparison, we compare percent increases of the ‘tooth crown size’ (square root of the calculated crown area [MD × BL]) from M_1_ to M_2_ ([M_2_−M_1_]/M_1_), and from M_2_ to M_3_ ([M_3_−M_2_]/M_2_). The results for the M_2_-M_3_ size proportions are reported in S3 Table and S1 Text because we found no clear inter-group differences for this trait.

As for the M_1_-M_2_ size proportion, the results in Table 2 show that *H*. *habilis* exhibits a primitive pattern of M_1_ < M_2_ (5−20% increase). Early Javanese *H*. *erectus* is also close to this primitive condition (4−9% increase), whereas the pattern observed in two *H*. *floresiensis* individuals, M_1_ ≈ M_2_ (0% [LB1] and 1% [LB6/1]), is found in Dmanisi *Homo*, *H*. *ergaster*, East Asian Middle Pleistocene archaic *Homo*, and *H*. *sapiens*. Thus, the M_1_-M_2_ size pattern exhibited by *H*. *floresiensis* appeared only after *H*. *habilis*. Our global *H*. *sapiens* sample shows a wide range of variation from −15% to +5% with a weak correlation with the crown size (M_1_ + M_2_) (r = 0.202). The values of the two *H*. *floresiensis* individuals are atypical for *H*. *sapiens* when this correlation is taken into consideration (Fig 7D).

#### Alveolar arcade shape (no. 23 in Table 2)

Alveolar arcade shape is a useful indicator in hominin taxonomy [30,33,92–94]. A previous study showed that the mandibular arcade shapes of LB1 and LB6/1 are broader (more derived) than *Au*. *afarensis* (L.H. 4; A.L. 266–1, 288-1i, 400-1a; MAK-VP-1/12), *H*. *habilis sensu lato* (KNM-ER 1805, OH13), and Dmanisi *Homo* (D211, 2600), narrower (more primitive) than most of the African post-*habilis* archaic *Homo* (KNM-ER 730; KNM-BK 8518; Tighenif 1, 3) and Chinese *H*. *erectus* (Zhoukoudian H1), but similar to the arcades of early Javanese *H*. *erectus* (Sangiran 9 and 22) [2].

Table 2 shows a data set cited from ref. [2] and including the index values from one recently discovered and two newly reconstructed *H*. *habilis s*. *l*. mandibles: KNM-ER 60000 (adult, 126%) [42], OH 7 (late juvenile, 156%) [30] and KNM-ER 1802 (adolescent, 147%) [30]. Here, the ‘alveolar arcade index’ is the length-breadth ratio based on the distance between the right and left intersection points of the distal contour of M_3_ and the midline of molar row (breadth), and the distance from infradentale to a line tangential to the distal faces of right and left M_3_ crowns (length). The inclusion of these additional specimens further strengthens the previous conclusion: the two *H*. *floresiensis* mandibles are derived compared to *H*. *habilis* and Dmanisi *Homo*, and are most similar to early Javanese *H*. *erectus* in mandibular arcade shape.

## Discussion

We comprehensively analyzed individual dental characters of *H*. *floresiensis* by combining various metric (linear measurement and EFA), other numerical, and statistical comparisons. Table 3 integrates the results of these analyses to summarize dental evolutionary trends in *Homo*. As for the categorical traits (#1-#23), primitive conditions exhibited by the *H*. *habilis sensu lato* sample are described in the “Character” list, and “yes” means that the relevant group is primitive for that trait. The “Status” column denotes morphological status of *H*. *floresiensis* as described in the footnote of this table. Table 3 is based on a large sample of the Early Pleistocene *Homo* (East Africa, Georgia, and Java) and a large, global sample of modern human teeth, and thus provides us with a reliable framework to evaluate the dental evolutionary position of *H*. *floresiensis* (and perhaps other *Homo* specimens from different regions).

**Table 3:** Dental character distribution in the *Homo* groups and status of *H*. *floresiensis*.

			EP				MP	LP		Status^a^
	Character	Refer to	*H*. *hab*.	*H*. *erg*.	Dmanisi	eJ*He*	E-Asia	*H*. *sap*.	*H*. *flo*.	*H*. *flo*.
#1	C^1^ distal shoulder low (vs. high)	1 in Table 2, Fig 7A	yes	−	−	yes	yes	no	yes	EP-MP
#2	P^3^ & P^4^ transverse crest often present	2 & 3 in Table 2	yes	yes?	?	yes	no?	no	yes	EP
#3	P^3^ buccal groove(s) always present	4 in Table 2	yes	no	no	no	no	no	no	post-Hh
#4	P^4^ crown buccal ≤ lingual	6 in Table 2	yes	?	yes	yes	no	no	yes	EP
#5	P_3_ crown long MD & asymmetric	PCs1 & 2 in Fig 4A	yes	yes	−	yes	−	no	yes	EP
#6	P_3_ lingual cusp located mesially (vs. distally on occasion)	7 in Table 2	yes	no	no	yes	no	no	no	post-Hh
#7	P_3_ mesiolingual crown normal (vs. beveled and wrinkled)	9 in Table 2	yes	yes	yes	yes	yes	yes	no	unique
#8	P_3_ buccal basal enamel swelling	14 in Table 2	yes	no	yes	yes	yes	no	yes	EP-MP
#9	P_3_ root often bifurcated (vs. Tomes' form or single)	15 in Table 2	yes	yes	yes?	yes	no	no	yes	EP
#10	P_4_ crown oblique (vs. MD symmetric)	PC2 in Fig 4C	yes	yes	−	yes	−	variable	yes	EP range
#11	P_4_ crown triangular (vs. more circular)	PC3 in Fig 4D	yes	variable	−	no	−	variable	no	post-Hh
#12	P_4_ transverse crest absent (vs. present)	11 in Table 2	yes	no	no	no	no	often yes	no	post-Hh
#13	M^1^ crown long & parallelogram (vs. short & rectangle)	PCs1 & 2 in Fig 5A	yes	yes	−	no	−	variable	no+	unique
#14	M^1^ crown distobuccal corner projected (vs. abbreviated)	PC3 in Fig 5B	yes	yes	−	yes	−	variable	yes	EP range
#15	M^2^ crown long MD and distally tapering	PCs1 & 4 in Fig 5C and 5D	yes	yes	−	yes	−	variable	yes	EP range
#16	M^2^ crown parallelogram (vs. inverted trapezoid)	PC2 in Fig 5C	yes	yes	−	no	−	variable	no	post-Hh
#17	M_1_ crown long MD (vs. short)	PC1 in Figs 6A and 7C	yes	yes	−	no	−	no	no+	unique
#18	M_1_ crown squarish (vs. pentagonal)	PC3 in Fig 6B	yes	variable	−	yes	−	variable	yes	EP range
#19	M_1_ five-cusped (vs. four-cusped)	17 in Table 2	yes	yes	yes	yes	yes	not always	no	Hs
#20	M_2_ crown long MD (vs. short)	PC1 in Fig 6C	yes	variable	−	no	−	variable	no	post-Hh
#21	M_2_ crown rounded (vs. square)	PC3 in Fig 6D	yes	yes	−	yes	−	variable	yes	EP range
#22	M_2_ five-cusped (vs. four-cusped or less)	18 in Table 2	yes	yes	yes	no?	yes	no	no	Hs?
#23	M_1_ mid-trigonid crest absent or rare	19 in Table 2	yes	?	no	no	?	yes	no	post-Hh
#24	Relative P_3_ size	21 in Table 2	moderate	moderate	moderate	moderate	moderate	small	large	unique
#25	M_1_/M_2_ size proportion	22 in Table 2, Fig 7D	M_1_ < M_2_	M_1_ ≤ M_2_	M_1_ ≤ M_2_	M_1_ < M_2_	M_1_ = M_2_	M_1_ ≥ M_2_	M_1_ = M_2_	post-Hh
#26	Alveolar arcade index (length-breadth index [%])	23 in Table 2	narrow	wide?	narrow	moderate	wide	−	moderate	eJHe

EP = Early Pleistocene; MP = Middle Pleistocene; LP = Late Pleistocene; *H*. *hab*. = *H*. *habilis*; *H*. *erg*. = *H*. *ergaster*; eJ*He* = early Javanese *H*. *erectus*; MP E-Asia = East Asian Middle Pleistocene archaic *Homo*; *H*. *sap*., Hs = *H*. *sapiens*; *H*. *flo*. = *H*. *floresiensis*.

^a^Status of *H*. *floresiensis* is categorized as follows: EP = a primitive condition shared with the Early Pleistocene *Homo*; EP-MP = a primitive condition shared with the Early-Middle Pleistocene *Homo*; EP range = within the range of the Early Pleistocene *Homo*, which is mostly encompassed by the large variation of *H*. *sapiens*; post-Hh = derived from the *H*. *habilis* condition; Hs = derived like *H*. *sapiens*; unique = unique among all the samples compared; eJHe = similar to the condition of early Javanese *H*. *erectus*.

Based on Table 3, we examined character distributions of the 26 dental traits in the various *Homo* groups compared here, and evaluated the dental evolutionary position of *H*. *floresiensis*. We did not employ here a cladistic or distance analyses. Although such methods can integrate information from different characters, the results would be difficult to interpret because we still do not have enough knowledge about at least two essential elements for these types of analyses: intercharacter correlations (i.e., some characters may be more or less correlated to each other and thus cannot be regarded as independent characters), and relative taxonomic significance for each character (i.e., some of these 26 traits may be more significant than others in taxonomic identification). We acknowledge that our “traditional” character list approach is also not free from these problems. Because of this, for example, simple counts of shared/unshared traits should not be directly translated as to the strength (or weakness) of taxonomic affinity between the groups under comparison. However, we found that the results of this analysis are consistent, straightforward, and clearly informs about dental morphological trends in *H*. *floresiensis*. Table 3 shows that *H*. *floresiensis* shares primitive conditions with some or all of the Early Pleistocene *Homo* groups in 20 traits (indicated as ‘EP,’ ‘EP-MP,’ ‘EP range,’ ‘post-Hh,’ or ‘eJHe’ in the Status column), unique in four traits (‘unique’ in the Status column), and derived like *H*. *sapiens* in two traits (‘Hs’ in the Status column).

We first discuss the 20 possibly primitive dental traits. Ten of these (nos. #3, #6, #10, #11, #14−16, #18, #20, #21) are also shared by *H*. *sapiens* due to the latter’s large variation [68] and may not be taxonomically diagnostic. Further, we do not have comparative data for the *H*. *sapiens* sample for another character (#26). However, in the other nine characters (nos. #1, #2, #4, #5, #8, #9, #12, #23, #25), *H*. *sapiens* is derived and different from *H*. *floresiensis* and other archaic *Homo* groups. This finding contradicts the previous suggestion that the dental morphology of *H*. *floresiensis* is entirely modern [23]. In sharp contrast to the lineage leading to *H*. *sapiens*, *H*. *floresiensis* retains many primitive dental morphologies despite being dramatically reduced in tooth size (Fig 2).

*H*. *floresiensis* is ‘unique’ in four traits. Two of them (nos. #7 and #24) are the large relative size and the unique occlusal morphology of the P_3_ that otherwise exhibits primitive morphologies (nos. #5, #8, and #9). The other two (nos. #13 and #17) primarily reflect their extremely short first molars. In view of the general trend of molar shortening during *Homo* evolution (Figs 5A and 6A), this highly derived condition in *H*. *floresiensis* may be described as “hyper-modern.”

*H*. *floresiensis* shares one characteristic feature of *H*. *sapiens*: the reduced cusp number on the M_1_ and M_2_ (from five to four: nos. #19 and #22). It should be noted that, however, this morphology was recently reported for some surface finds from Sangiran, which are tentatively included in the chronologically younger (Sangiran Upper) subgroup of early Javanese *H*. *erectus* [35]. Because this reduction is correlated with reduced molar size within modern human samples (see “M_1_ cusp number” and “M_2_ cusp number” above), it is possible that these “modern” morphologies are all associated with the crown shortening (nos. #13 and #17).

Overall, our analyses demonstrate that the dentition of *H*. *floresiensis* exhibits a unique combination of primitive traits on the canine-premolars and some modern or even hyper-modern traits on the molars.

As for the primitive dental characters seen in *H*. *floresiensis*, these are either those widely shared among different Early Pleistocene taxa (*H*. *habilis*, *H*. *ergaster*, Dmanisi *Homo*, early Javanese *H*. *erectus*) (nos. #1, #2, #4, #5, #8‒10, #14, #15, #18, #21), or characters that appeared after *H*. *habilis* (nos. #3, #6, #11, #12, #16, #20, #23, #25, #26). Importantly, none of them exhibit very primitive conditions restricted to *H*. *habilis*. Thus, the dental morphology of *H*. *floresiensis* is derived relative to *H*. *habilis s*. *l*. and is comparable to post-*habilis* grade Early Pleistocene *Homo* or *H*. *erectus s*. *l*. Size-related morphological changes from the *H*. *habilis* condition, if any, do not explain the observed resemblance between *H*. *erectus s*. *l*. and *H*. *floresiensis* because their tooth sizes greatly differ from each other (Fig 2).

It has been claimed that the brain size of *H*. *floresiensis* is far too small to be attributed to intraspecific dwarfism from *H*. *erectus*, but recent studies showed that the decrease of human brain size associated with body size reduction [25] as well as the decrease of relative brain size in insular primates [95] could be substantial. Some researchers found affinities with earlier African hominins in the mandibles and other skeletal elements of *H*. *floresiensis* [14–19], but these remain highly controversial [2,20]. Now, the evidence from skull [2,3] and teeth (this study), the most diagnostic elements in evolutionary systematics of the genus *Homo*, converge on the same conclusion. Combined with other evidence such as geographic proximity and a report that the earliest evidence for hominins on Flores (~1.0 million years ago) [26] does not exceed the oldest record for *H*. *erectus* in Java (≥1.2 million years ago) [31,32], we suggest that *H*. *floresiensis* evolved from early Javanese *H*. *erectus* or a related form from the ancient Sundaland, whose absolute brain size was about twice as large [25]. *H*. *floresiensis* is not evidence for unexpectedly early hominin dispersal into Asia [14–19], but is more likely an example of considerably greater flexibility in hominin physical evolution as originally proposed [1,2,20,21].

Finally, the reduced first molar of *H*. *floresiensis* is of interest. The first molar is one of the most invariant human teeth [96] and its size and morphology have been relatively conservative in hominin dental evolution [56], but this is the tooth that is most markedly shortened in the dentition of *H*. *floresiensis* (Fig 2). The reason why *H*. *floresiensis* experienced such a unique evolution is unclear. It is often supposed that dietary changes and food-processing practices that decrease chewing stress were the prime movers of the molar size reduction in the Pleistocene *Homo* [56,91,97,98]. Was this also the case for the first molar reduction in *H*. *floresiensis*? This hypothesis [9] may not be unrealistic in view of their relatively small facial skeleton [2,9], and a recent biomechanical study that concluded that their mandibles (LB1 and LB6/1) did not recruit masticatory forces on the order of what is utilized by, at least, South African australopiths [9]. However, evidence for advanced food-processing is currently not recognized at least in their simple stone technology [99], and the diets of *H*. *floresiensis* still remain to be studied [100]. Further archaeological and other research programs are needed to address this interesting question.

## Conclusions

Our comprehensive comparative analyses of the teeth of *H*. *floresiensis* indicated that they are primitive relative to *H*. *sapiens* in displaying a low C_1_ distal shoulder, P^3^ and P^4^ transverse crests, a well-developed P^4^ lingual crown, a MD long and asymmetric P_3_ crown, a P_3_ buccal basal enamel swelling (cingulum), a bifurcated P_3_ root, a P_4_ transverse crest, a M_1_ mid-trigonid crest, and equivalent M_1_ and M_2_ sizes. The abundance of such primitive features contradicts the claim that *H*. *floresiensis* is not a new species but belongs to *H*. *sapiens* [23].

*H*. *floresiensis* share a number of dental characteristics with Early Pleistocene *Homo*, but none of them exhibit very primitive conditions restricted to *H*. *habilis sensu lato* (here defined as East African *Homo* specimens earlier than 1.75 million years ago). Instead, many of these primitive features are derived features that *H*. *floresiensis* shares with one or more post-*habilis* grade Early Pleistocene *Homo* from East Africa (*H*. *ergaster*), Java (early Javanese *H*. *erectus*), and Georgia (Dmanisi *Homo*). Such features include the occasional absence of a P^3^ buccal groove, a distally positioned P_3_ lingual cusp, a more circular P_4_ crown, the presence of a P_4_ transverse crest, non-parallelogram M^2^ crown shape, a MD short M_2_ crown, a M_1_ mid-trigonid crest, equivalent M_1_ and M_2_ sizes, and a moderately wide alveolar arcade. Therefore, dental morphology does not support the previous claim that *H*. *floresiensis* evolved from a form of hominin more primitive than *H*. *erectus*, such as *H*. *habilis* or *Australopithecus* [14–19].

The analyses of the crown contours of the six premolars and molars indicated that *H*. *floresiensis* is more similar to early Javanese *H*. *erectus* rather than to African *H*. *ergaster* or *H*. *habilis*. Although more detailed comparisons with Dmanisi *Homo* have yet to be conducted, the above results give additional, strong support to the hypothesis that *H*. *floresiensis* evolved from an early Javanese *H*. *erectus* population or a related form from the ancient Sundaland with substantial body and brain size dwarfism [1,2,20,25]. There are also several unique dental features in *H*. *floresiensis* (P_3_ morphology, extremely short M^1^ and M_1_, and generally primitive canine-premolar vs. progressive molar morphologies) whose functional significance remains to be investigated.

## Supporting Information

S1 Fig Examples of the “isolated” dental casts used for the present study. (TIF)

S2 Fig The system used for photography of the dental specimens for crown counter extraction. The photographic system (A) and the specimen table (B). A 100 mm macro lens was set to a Canon D40 digital camera to minimize the parallax effect. For ease and accuracy in orienting the teeth, a special camera stand equipped with a horizontally movable turning table was used: Each dental cast was placed on the table using modeling clay, and the cervical plane was determined by turning the table, which was then moved horizontally so that the tooth was placed immediately below the camera lens. For the calibration in a later step, a millimeter scale was inserted to each photograph at the level of the deepest point on the occlusal grooves, using a leveling devise to control the scale’s horizontal orientation. As far as possible, the background of the dental cast was made dark by putting black sticky tapes.
(TIF)

S3 Fig Steps of dental calculus elimination and crown contour extraction for the three LB1 molars. Calculus was identified (red) (A), and removed virtually (B) in the micro-CT imagery. Calculus was clearly distinguishable from the enamel as parts with lesser CT values, as seen in horizontal CT sections (C). A digital photographic image of the high-quality plaster cast was prepared for each tooth, and its background was removed semi-automatically using an image-processing software (D). Then, the calculus was deleted by superimposing the image of B onto D, and worn parts of the tooth were reconstructed with reference to the 3D topography of the plaster cast (blue) to prepare the final image for contour extraction (E).
(TIF)

S1 Table Comparative Early Pleistocene *Homo* samples. (PDF)

S2 Table Comparative *Homo sapiens* sample. (PDF)

S3 Table Results of the non-metric and linear metric comparisons. (PDF)

S4 Table Linear metric and non-metric data used in this study. (XLSX)

S1 Text Additional notes on the non-metric and linear metric comparisons. (PDF)

## References

[1] Brown P, Sutikna T, Morwood MJ, Soejono RP, Jatmiko, Wahyu Saptomo E, et al A new small-bodied hominin from the Late Pleistocene of Flores, Indonesia. Nature. 2004; 431: 1055–1061. 15514638 10.1038/nature02999

[2] Kaifu Y, Baba H, Sutikna T, Morwood MJ, Kubo D, Wahyu Saptomo E, et al Craniofacial morphology of *Homo floresiensis*: description, taxonomic affinities, and evolutionary implication. J Hum Evol. 2011; 61: 644–682. 10.1016/j.jhevol.2011.08.008 22036083

[3] Baab KL, McNulty KP, Harvati K. *Homo floresiensis* contextualized: a geometric morphometric comparative analysis of fossil and pathological human samples. PLoS ONE. 2013; 8: e69119 10.1371/journal.pone.0069119 23874886 PMC3707875

[4] Morwood MJ, Brown P, Jatmiko, Sutikna T, Saptomo EW, Westaway KE, et al Further evidence for small-bodied hominins from the Late Pleistocene of Flores, Indonesia. Nature. 2005; 437: 1012–1017. 16229067 10.1038/nature04022

[5] Argue D, Donlon D, Groves C, Wright R. *Homo floresiensis*: microcephalic, pygmoid, Australopithecus, or Homo? J Hum Evol. 2006; 51: 360–374. 16919706 10.1016/j.jhevol.2006.04.013

[6] Jungers WL. Interlimb proportions in humans and fossil hominins: variability and scaling In: Grine FE, Leakey RE, Fleagle JG, editors. The First Humans: Origins of the Genus *Homo*. Dordrecht: Springer; 2009 pp. 93–98.

[7] Holliday TW, Franciscus RG. Humeral length allometry in African hominids (sensu lato) with special reference to A.L. 288–1 and Liang Bua 1. PaleoAnthropol. 2012; 2012: 1–12.

[8] Falk D, Hildebolt C, Smith K, Morwood MJ, Sutikna T, Jatmiko, et al LB1's virtual endocast, microcephaly, and hominin brain evolution. J Hum Evol. 2009; 57: 597–607. 10.1016/j.jhevol.2008.10.008 19254807

[9] Daegling DJ, Patel BA, Jungers WL. Geometric properties and comparative biomechanics of *Homo floresiensis* mandibles. J Hum Evol. 2014; 68: 36–46. 10.1016/j.jhevol.2014.01.001 24560803

[10] Tocheri MW, Orr CM, Larson SG, Sutikna T, Jatmiko, Wahyu Saptomo E, et al The primitive wrist of *Homo floresiensis* and its implications for hominin evolution. Science. 2007; 317: 1743–1745. 17885135 10.1126/science.1147143

[11] Orr CM, Tocheri MW, Burnett SE, Awe RD, Saptomo EW, Sutikna T, et al New wrist bones of *Homo floresiensis* from Liang Bua (Flores, Indonesia). J Hum Evol. 2013; 64: 109–129. 10.1016/j.jhevol.2012.10.003 23290261

[12] Larson SG, Jungers WL, Tocheri MW, Orr CM, Morwood MJ, Sutikna T, et al Descriptions of the upper limb skeleton of *Homo floresiensis* . J Hum Evol. 2009; 57: 555–570. 10.1016/j.jhevol.2008.06.007 19056103

[13] Jungers WL, Larson SG, Harcourt-Smith W, Morwood MJ, Sutikna T, Rokus Due Awe et al Descriptions of the lower limb skeleton of *Homo floresiensis* . J Hum Evol. 2009; 57: 538–554. 10.1016/j.jhevol.2008.08.014 19062072

[14] Jungers WL, Harcourt-Smith WE, Wunderlich RE, Tocheri MW, Larson SG, Sutikna T, et al The foot of *Homo floresiensis* . Nature. 2009; 459: 81–84. 10.1038/nature07989 19424155

[15] Brown P, Maeda T. Liang Bua *Homo floresiensis* mandibles and mandibular teeth: a contribution to the comparative morphology of a new hominin species. J Hum Evol. 2009; 57: 571–596. 10.1016/j.jhevol.2009.06.002 19589559

[16] Jungers WL. Homo floresiensis In: Begun DR, editor. A Companion to Paleoanthropology. New Jersey: Blackwell Publishing Ltd; 2013 pp. 582–598.

[17] Morwood MJ, Jungers WL. Conclusions: implications of the Liang Bua excavations for hominin evolution and biogeography. J Hum Evol. 2009; 57: 640–648. 10.1016/j.jhevol.2009.08.003 19913680

[18] Gordon AD, Nevell L, Wood B. The *Homo floresiensis* cranium (LB1): size, scaling, and early *Homo* affinities. Proc Natl Acad Sci USA. 2008; 105: 4650–4655. 10.1073/pnas.0710041105 18356300 PMC2290802

[19] Argue D, Morwood MJ, Sutikna T, Jatmiko, Saptomo EW. *Homo floresiensis*: a cladistic analysis. J Hum Evol. 2009; 57: 623–639. 10.1016/j.jhevol.2009.05.002 19628252

[20] van Heteren AH. The hominins of Flores: Insular adaptations of the lower body. Compt Rendu Palevol. 2012; 11: 169–179.

[21] Lyras GA, Dermitzakis MD, Van Der Geer AAE, Van Der Geer SB, De Vos J. The origin of *Homo floresiensis* and its relation to evolutionary processes under isolation. Anthropol Sci. 2009; 117: 33–43.

[22] Bailey SE, Hublin JJ, editors. Dental Perspectives on Human Evolution: State of the Art Research in Dental Paleoanthropology Dordrecht: Springer; 2007.

[23] Jacob T, Indriati E, Soejono RP, Hsu K, Frayer DW, Eckhardt RB, et al Pygmoid Australomelanesian *Homo sapiens* skeletal remains from Liang Bua, Flores: population affinities and pathological abnormalities. Proc Natl Acad Sci USA. 2006; 103: 13421–13426. 16938848 10.1073/pnas.0605563103 PMC1552106

[24] Kaifu Y, Kono RT, Sutikna T, Saptomo EW, Jatmiko, Rokus Due Awe, et al Descriptions of the dental remains of *Homo floresiensis* . Anthropol Sci. 2015; 123: 129–145. 10.1371/journal.pone.0141614 PMC4651360 26624612

[25] Kubo D, Kono RT, Kaifu Y. Brain size of *Homo floresiensis* and its evolutionary implications. Proc R Soc B. 2013; 280: 20130338 10.1098/rspb.2013.0338 23595271 PMC3652458

[26] Brumm A, Jensen GM, van den Bergh GD, Morwood MJ, Kurniawan I, Aziz F, et al Hominins on Flores, Indonesia, by one million years ago. Nature. 2010; 464: 748–752. 10.1038/nature08844 20237472

[27] Lordkipanidze D, Ponce de Leon M, Margvelashvili A, Rak Y, Rightmire GP, Vekua A, et al A complete skull from Dmanisi, Georgia, and the evolutionary biology of early *Homo* . Science. 2013; 342: 326–331. 10.1126/science.1238484 24136960

[28] Wood B. Fifty years after *Homo habilis* . Nature. 2014; 508: 31–33. 24707524 10.1038/508031a

[29] Anton SC, Potts R, Aiello LC. Human evolution. Evolution of early *Homo*: an integrated biological perspective. Science. 2014; 345: 1236828 10.1126/science.1236828 24994657

[30] Spoor F, Gunz P, Neubauer S, Stelzer S, Scott N, Kwekason A, et al Reconstructed *Homo habilis* type OH 7 suggests deep-rooted species diversity in early *Homo* . Nature. 2015; 519: 83–86. 10.1038/nature14224 25739632

[31] Hyodo M, Matsu'ura S, Kamishima Y, Kondo M, Takeshita Y, Kitaba I, et al High-resolution record of the Matuyama-Brunhes transition constrains the age of Javanese *Homo erectus* in the Sangiran dome, Indonesia. Proc Natl Acad Sci USA. 2011; 108: 19563–19568. 10.1073/pnas.1113106108 22106291 PMC3241771

[32] Larick R, Ciochon RL, Zaim Y, Sudijono, Suminto, Rizal Y, et al Early Pleistocene 40Ar/39Ar ages for Bapang Formation hominins, Central Jawa, Indonesia. Proc Natl Acad Sci USA. 2001; 98: 4866–4871. 11309488 10.1073/pnas.081077298 PMC33129

[33] Kaifu Y, Baba H, Aziz F, Indriati E, Schrenk F, Jacob T. Taxonomic affinities and evolutionary history of the Early Pleistocene hominids of Java: dentognathic evidence. Am J Phys Anthropol. 2005; 128: 709–726. 15761880 10.1002/ajpa.10425

[34] Kaifu Y. Advanced dental reduction in Javanese *Homo erectus* . Anthropol. Sci. 2006; 114: 35–43.

[35] Zanolli C. Additional evidence for morpho-dimensional tooth crown variation in a New Indonesian *H*. *erectus* sample from the Sangiran Dome (Central Java). PLoS ONE. 2013; 8: e67233 10.1371/journal.pone.0067233 23843996 PMC3700995

[36] Kaifu Y, Indriati E, Aziz F, Kurniawan I, Baba H. Cranial Morphology and Variation of the Earliest Indonesian Hominids In: Norton CJ, Braun DR, editors. Asian Paleoanthropology: From Africa to China and Beyond. Dordrecht: Springer; 2010 pp. 143–157.

[37] Kaifu Y, Zaim Y, Baba H, Kurniawan I, Kubo D, Rizal Y, et al New reconstruction and morphological description of a *Homo erectus* cranium: skull IX (Tjg-1993.05) from Sangiran, Central Java. J Hum Evol. 2011; 61: 270–294. 10.1016/j.jhevol.2011.04.002 21683428

[38] Aziz F, Baba H, editors. *Homo erectus* in Indonesia Recent Progress of the Study and Current Understanding. Bandung: Centre for Geological Survey; 2013.

[39] Bae CJ. The late Middle Pleistocene hominin fossil record of eastern Asia: synthesis and review. Yrb Phys Anthropol. 2010; 53: 75–93. 10.1002/ajpa.21442 21086528

[40] Suwa G. A comparative analysis of hominid dental remains from the Sungura and Usno Formations, Omo Valley, Ethiopia. PhD dissertation, University of California at Berkeley; 1990.

[41] Wood B. Koobi Fora Research Project 4: Hominid Cranial Remains Oxford: Clarendon Press; 1991.

[42] Leakey MG, Spoor F, Dean MC, Feibel CS, Anton SC, Kiarie C, et al New fossils from Koobi Fora in northern Kenya confirm taxonomic diversity in early *Homo* . Nature. 2012; 488: 201–204. 10.1038/nature11322 22874966

[43] Martinón-Torres M, Bermúdez de Castro JM, Gómez-Robles A, Margvelashvili A, Prado L, Lordkipanidze D, et al Dental remains from Dmanisi (Republic of Georgia): morphological analysis and comparative study. J Hum Evol. 2008; 55: 249–273. 10.1016/j.jhevol.2007.12.008 18486183

[44] Brown B, Walker A. The dentition In: Walker A, Leakey R, editors. The Nariokotome *Homo erectus* skeleton. Cambridge, MA: Harvard Univesity Press; 1993 pp. 161–192.

[45] Weidenreich F. The dentition of Sinanthropus pekinensis: a comparative odontography of the hominids. Palaeontol Sin New Ser D. 1937; 1: 1–180.

[46] Xing S, Martinón-Torres M, Bermúdez de Castro JM, Wu X, Liu W. Hominin teeth from the early Late Pleistocene site of Xujiayao, Northern China. Am J Phys Anthropol. 2015; 56: 224–240. 10.1002/ajpa.22641 25329008

[47] Kuhl FP. Elliptic Fourier features of a closed contour. Comput Graph Image Process. 1982; 18: 236–258.

[48] Lestrel PE, Wolfe CA, Bodt A. Mandibular shape analysis in fossil hominins: Fourier descriptors in norma lateralis. Homo. 2013; 64: 247–272. 10.1016/j.jchb.2013.05.001 23769600

[49] Gómez-Robles A, Martinón-Torres M, Bermúdez de Castro JM, Prado-Simon L, Arsuaga JL. A geometric morphometric analysis of hominin upper premolars. Shape variation and morphological integration. J Hum Evol. 2011; 61: 688–702. 10.1016/j.jhevol.2011.09.004 22047673

[50] Wood B, Abbott SA. Analysis of the dental morphology of Plio-Pleistocene hominids. I. Mandibular molars: crown area measurements and morphological traits. Journal of Anatomy. 1983; 136: 197–219. 6403498 PMC1171940

[51] Bailey SE, Lynch JM. Diagnostic differences in mandibular P4 shape between Neandertals and anatomically modern humans. Am J Phys Anthropol. 2005; 126: 268–277. 15386225 10.1002/ajpa.20037

[52] Martinón-Torres M, Bastir M, Bermúdez de Castro JM, Gomez A, Sarmiento S, Muela A, et al Hominin lower second premolar morphology: evolutionary inferences through geometric morphometric analysis. J Hum Evol. 2006; 50: 523–533. 16472839 10.1016/j.jhevol.2005.12.004

[53] Iwata H, Ukai Y. SHAPE: A computer program package for quantitative evaluation of biological shapes based on elliptic Fourier descriptors. Journal of Heredity. 2002; 93: 384–385. 12547931 10.1093/jhered/93.5.384

[54] Abdi H. Bonferroni test In: Salkind NJ, editor. Encyclopedia of Measurement and Statistics. Thousand Oaks, CA: Sage; 2007 pp. 103–106.

[55] Robinson JT. The Dentition of the Australopithecinae. Pretoria: Transvaal Museum; 1956.

[56] Wolpoff MH. Metric Trends in Hominid Dental Evolution. Cleveland: Case Western Reserve University; 1971.

[57] Wood BA, Abbott SA, Graham SH. Analysis of the dental morphology of Plio-Pleistocene hominids. II. Mandibular molars—study of cusp areas, fissure pattern and cross sectional shape of the crown. J Anat. 1983; 137: 287–314. 6415025 PMC1171822

[58] Wood B, Uytterschaut H. Analysis of the dental morphology of Plio-Pleistocene hominids. III. Mandibular premolar crowns. J Anat. 1987; 154: 121–156. 3128512 PMC1261842

[59] Wood B, Engleman CA. Analysis of the dental morphology of Plio-Pleistocene hominids V. Maxillary postcanine tooth morphology. J Anat. 1988; 161: 1–35. 3254883 PMC1262088

[60] Wood BA, Abbott SA, Uytterschaut H. Analysis of the dental morphology of Plio-Pleistocene hominids. IV. Mandibular postcanine root morphology. J Anat. 1988; 156: 107–139. 3047096 PMC1261917

[61] Suwa G, White TD, Howell FC. Mandibular postcanine dentition from the Shungura Formation, Ethiopia: crown morphology, taxonomic allocations, and Plio-Pleistocene hominid evolution. Am J Phys Anthropol. 1996; 101: 247–282. 8893088 10.1002/(SICI)1096-8644(199610)101:2<247::AID-AJPA9>3.0.CO;2-Z

[62] Tobias PV. Olduvai Gorge, 4: the skulls, endocasts and teeth of Homo habilis Cambridge: Cambridge University Press; 1991.

[63] Irish JD, Guatelli-Steinberg D. Ancient teeth and modern human origins: An expanded comparison of African Plio-Pleistocene and recent world dental samples. J Hum Evol. 2003; 45: 113–144. 14529648 10.1016/s0047-2484(03)00090-3

[64] Martinón-Torres M, Bermúdez de Castro JM, Gómez-Robles A, Arsuaga JL, Carbonell E, Lordkipanidze D, et al Dental evidence on the hominin dispersals during the Pleistocene. Proc Natl Acad Sci USA. 2007; 104: 13279–13282. 17684093 10.1073/pnas.0706152104 PMC1948952

[65] Martinón-Torres M, Bermúdez de Castro JM, Gómez-Robles A, Prado-Simón L, Arsuaga JL. Morphological description and comparison of the dental remains from Atapuerca-Sima de los Huesos site (Spain). J Hum Evol. 2012; 62: 7–58. 10.1016/j.jhevol.2011.08.007 22118969

[66] Bailey SE, Weaver TD, Hublin JJ. Who made the Aurignacian and other early Upper Paleolithic industries? J Hum Evol. 2009; 57: 11–26. 10.1016/j.jhevol.2009.02.003 19476971

[67] Bailey SE, Skinner MM, Hublin JJ. What lies beneath? An evaluation of lower molar trigonid crest patterns based on both dentine and enamel expression. Am J Phys Anthropol. 2011; 145: 505–518. 10.1002/ajpa.21468 21312178

[68] Bailey SE, Hublin J-J. What does it mean to be dentally "modern"? In: Scott GR, Irish JD, editors. Anthropological Perspectives on Tooth Morphology: Genetics, Evolution, Variation. Cambridge: Cambridge Univ. Press; 2013 pp. 222–249.

[69] Martínez de Pinillos M, Martinón-Torres M, Skinner MM, Arsuaga JL, Gracia-Téllez A, Martínez I, et al Trigonid crests expression in Atapuerca-Sima de los Huesos lower molars: Internal and external morphological expression and evolutionary inferences. Compt Rendu Palevol. 2014; 13: 205–221.

[70] Matsumura H, Hudson MJ. Dental perspectives on the population history of Southeast Asia. Am J Phys Anthropol. 2005; 127: 182–209. 15558609 10.1002/ajpa.20067

[71] Gómez-Robles A, Bermúdez de Castro JM, Martinón-Torres M, Prado-Simón L, Arsuaga JL. A geometric morphometric analysis of hominin lower molars: Evolutionary implications and overview of postcanine dental variation. J Hum Evol. 2015; 82: 34–50. 10.1016/j.jhevol.2015.02.013 25840859

[72] Gómez-Robles A, Bermúdez de Castro JM, Martinón-Torres M, Prado-Simon L, Arsuaga JL. A geometric morphometric analysis of hominin upper second and third molars, with particular emphasis on European Pleistocene populations. J Hum Evol. 2012; 63: 512–526. 10.1016/j.jhevol.2012.06.002 22840714

[73] Shields ED. Mandibular premolar and second molar root morphological variation in modern humans: What root number can tell us about tooth morphogenesis. Am J Phys Anthropol. 2005; 128: 299–311. 15838835 10.1002/ajpa.20110

[74] Johanson DC, White TD, Coppens Y. Dental remains from the Hadar Formation, Ethiopia: 1974–1 977 collections. Am J Phys Anthropol. 1982; 57: 545–603.

[75] Kimbel WH, Johanson DC, Rak Y. Systematic assessment of a maxilla of *Homo* from Hadar, Ethiopia. Am J Phys Anthropol. 1997; 103: 235–262. 9209580 10.1002/(SICI)1096-8644(199706)103:2<235::AID-AJPA8>3.0.CO;2-S

[76] Moggi-Cecchi J, Grine FE, Tobias PV. Early hominid dental remains from Members 4 and 5 of the Sterkfontein Formation (1966–1996 excavations): catalogue, individual associations, morphological descriptions and initial metrical analysis. J Hum Evol. 2006; 50: 239–328. 16309732 10.1016/j.jhevol.2005.08.012

[77] Delezene LK, Kimbel WH. Evolution of the mandibular third premolar crown in early *Australopithecus* . J Hum Evol. 2011; 60: 711–730. 10.1016/j.jhevol.2011.01.006 21481921

[78] Ward CV, Leakey MG, Walker A. Morphology of *Australopithecus anamensis* from Kanapoi and Allia Bay, Kenya. J Hum Evol. 2001; 41: 255–368. 11599925 10.1006/jhev.2001.0507

[79] Ward CV, Manthi FK, Plavcan JM. New fossils of *Australopithecus anamensis* from Kanapoi, West Turkana, Kenya (2003–2008). J Hum Evol. 2013; 65: 501–524. 10.1016/j.jhevol.2013.05.006 23998457

[80] Haile-Selassie Y, Melillo SM. Middle Pliocene hominin mandibular fourth premolars from Woranso-Mille (Central Afar, Ethiopia). J Hum Evol. 2015; 78: 44–59. 10.1016/j.jhevol.2014.08.005 25200889

[81] Wu X, Poirier FE. Human Evolution in China Oxford: Oxford University Press; 1995.

[82] Chang CH, Kaifu Y, Takai M, Kono RT, Grun R, Matsu'ura S, et al The first archaic *Homo* from Taiwan. Nat Commun. 2015; 6: 6037 10.1038/ncomms7037 25625212 PMC4316746

[83] Kupczik K, Hublin JJ. Mandibular molar root morphology in Neanderthals and Late Pleistocene and recent *Homo sapiens* . J Hum Evol. 2010; 59: 525–541. 10.1016/j.jhevol.2010.05.009 20719359

[84] Emonet EG, Tafforeau P, Chaimanee Y, Guy F, de Bonis L, Koufos G, et al Three-dimensional analysis of mandibular dental root morphology in hominoids. J Hum Evol. 2012; 62: 146–154. 10.1016/j.jhevol.2011.11.011 22189427

[85] Le Cabec A, Kupczik K, Gunz P, Braga J, Hublin JJ. Long anterior mandibular tooth roots in Neanderthals are not the result of their large jaws. J Hum Evol. 2012; 63: 667–681. 10.1016/j.jhevol.2012.07.003 23000085

[86] Xing S, Martinón-Torres M, Bermúdez de Castro JM, Zhang Y, Fan X, Zheng L, et al Middle Pleistocene hominin teeth from Longtan Cave, Hexian, China. PLoS ONE. 2014; 9(12): e114265 10.1371/journal.pone.0114265 25551383 PMC4281145

[87] Scott GR, Turner CG. The Anthropology of Modern Human Teeth. Cambridge: Cambridge University Press; 1997.

[88] Zanolli C. Molar crown inner structural organization in Javanese *Homo erectus* . Am J Phys Anthropol. 2015, 156: 148–157. 10.1002/ajpa.22611 25209431

[89] Bailey SE. A closer look at Neanderthal postcanine dental morphology: the mandibular dentition. Anat Rec. 2002; 269: 148–156. 12124901 10.1002/ar.10116

[90] Zanolli C, Bondioli L, Coppa A, Dean CM, Bayle P, Candilio F, et al The late Early Pleistocene human dental remains from Uadi Aalad and Mulhuli-Amo (Buia), Eritrean Danakil: macromorphology and microstructure. J Hum Evol. 2014; 74: 96–113. 10.1016/j.jhevol.2014.04.005 24852385

[91] Bermúdez de Castro JM, Nicolas ME. Posterior dental size reduction in hominids: the Atapuerca evidence. Am J Phys Anthropol. 1995; 96: 335–356. 7604890 10.1002/ajpa.1330960403

[92] Aiello L, Dean C. An Introduction to Human Evolutionary Anatomy. New York: Academic Press; 1990.

[93] Rosas A, Bermúdez de Castro JM. On the taxonomic affinities of the Dmanisi mandible (Georgia). Am J Phys Anthropol. 1998; 107: 145–162. 9786330 10.1002/(SICI)1096-8644(199810)107:2<145::AID-AJPA2>3.0.CO;2-U

[94] Villmoare B, Kimbel WH, Seyoum C, Campisano CJ, DiMaggio EN, Rowan J, et al Early *Homo* at 2.8 Ma from Ledi-Geraru, Afar, Ethiopia. Science. 2015; 347: 1352–1355. 10.1126/science.aaa1343 25739410

[95] Montgomery SH. Primate brains, the 'island rule' and the evolution of *Homo* floresiensis. J Hum Evol. 2013; 65: 750–760. 10.1016/j.jhevol.2013.08.006 24134961

[96] Kieser JA. Human Adult Odontometrics. Cambridge: Cambridge University Press; 1990.

[97] Brace CL, Rosenberg KR, Hunt KD. Gradual change in human tooth size in the Late Pleistocene and post-Pleistocene. Evolution. 1987; 41: 705–720. 28564360 10.1111/j.1558-5646.1987.tb05847.x

[98] Organ C, Nunn CL, Machanda Z, Wrangham RW. Phylogenetic rate shifts in feeding time during the evolution of *Homo* . Proc Natl Acad Sci USA. 2011; 108: 14555–14559. 10.1073/pnas.1107806108 21873223 PMC3167533

[99] Moore MW, Sutikna T, Jatmiko, Morwood MJ, Brumm A. Continuities in stone flaking technology at Liang Bua, Flores, Indonesia. J Hum Evol. 2009; 57: 503–526. 10.1016/j.jhevol.2008.10.006 19361835

[100] van den Bergh GD, Meijer HJ, Due Awe R, Morwood MJ, Szabo K, van den Hoek Ostende LW, et al The Liang Bua faunal remains: a 95k.yr. sequence from Flores, East Indonesia. J Hum Evol. 2009; 57: 527–537. 10.1016/j.jhevol.2008.08.015 19058833

