# Supplementary figures and images for: Unique Dental Morphology of Homo floresiensis and Its Evolutionary Implications
Source: PLoS One. 2015 Nov 18;10(11):e0141614. doi: 10.1371/journal.pone.0141614 (PMC4651360; doi:10.1371/journal.pone.0141614)

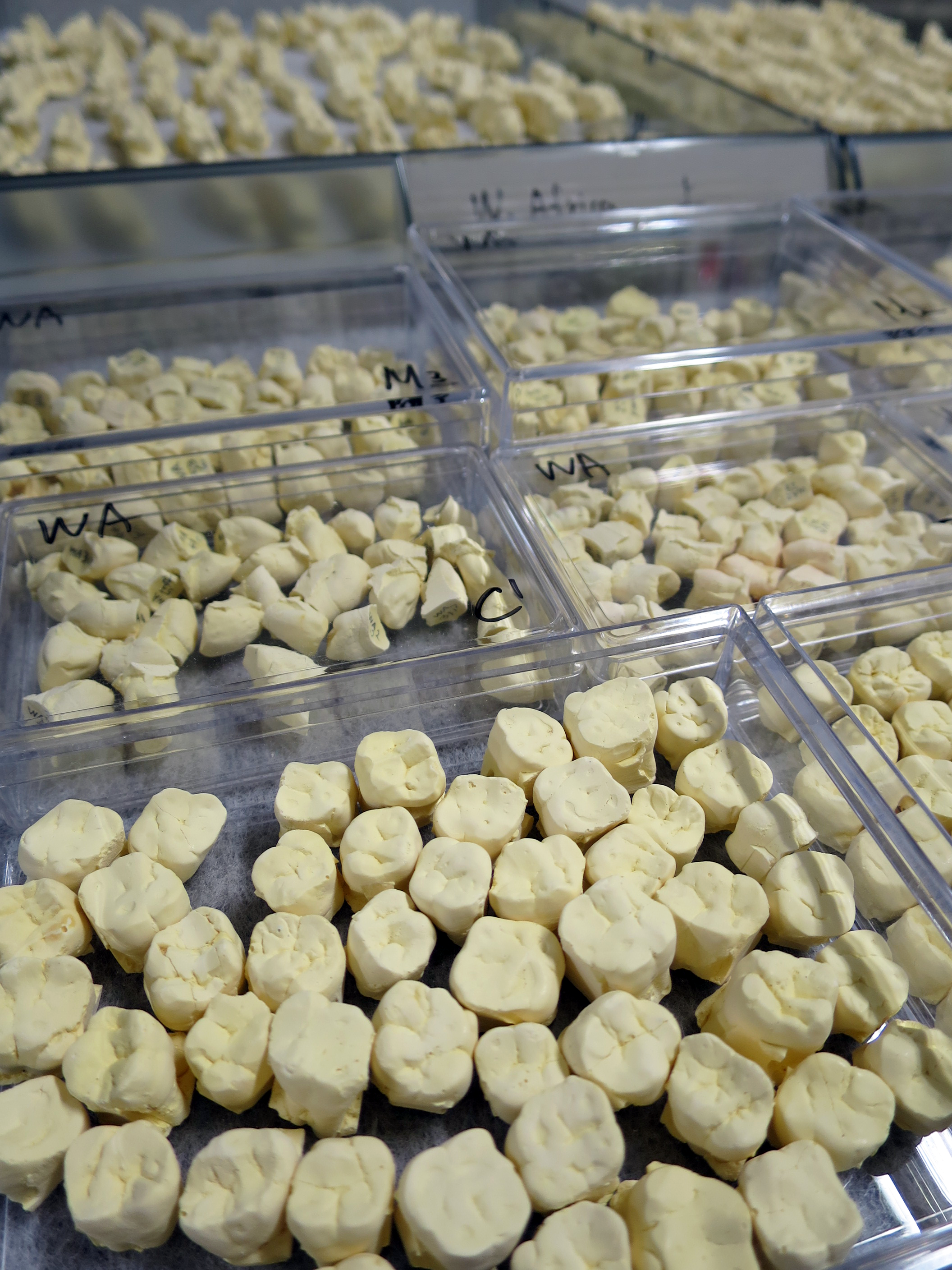

Supplement: S1 Fig — (TIF) [file pone.0141614.s001.tif]

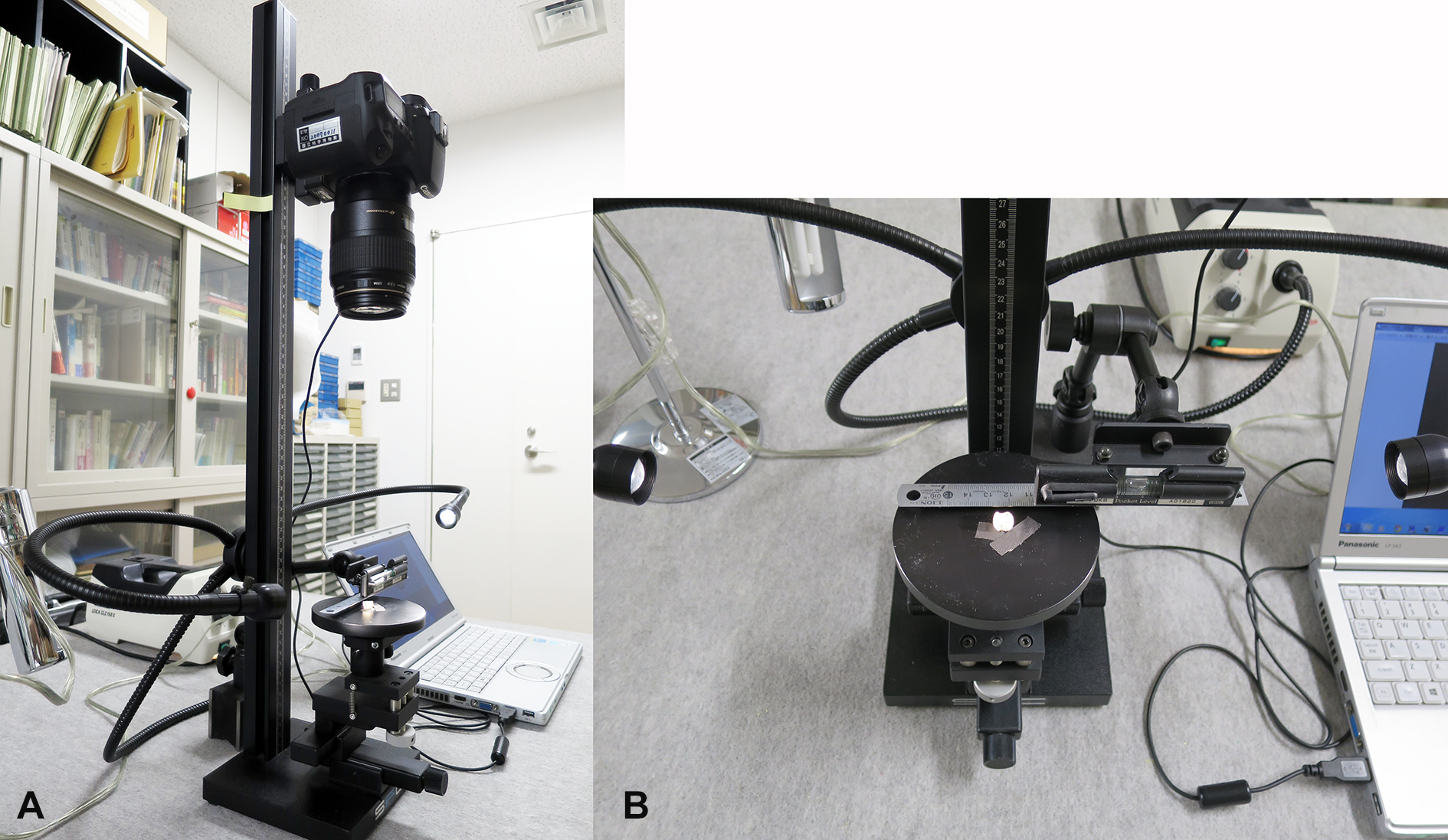

Supplement: S2 Fig — The photographic system (A) and the specimen table (B). A 100 mm macro lens was set to a Canon D40 digital camera to minimize the parallax effect. For ease and accuracy in orienting the teeth, a special camera stand equipped with a horizontally movable turning table was used: Each dental cast was placed on the table using modeling clay, and the cervical plane was determined by turning the table, which was then moved horizontally so that the tooth was placed immediately below the camera lens. For the calibration in a later step, a millimeter scale was inserted to each photograph at the level of the deepest point on the occlusal grooves, using a leveling devise to control the scale’s horizontal orientation. As far as possible, the background of the dental cast was made dark by putting black sticky tapes. (TIF) [file pone.0141614.s002.tif]

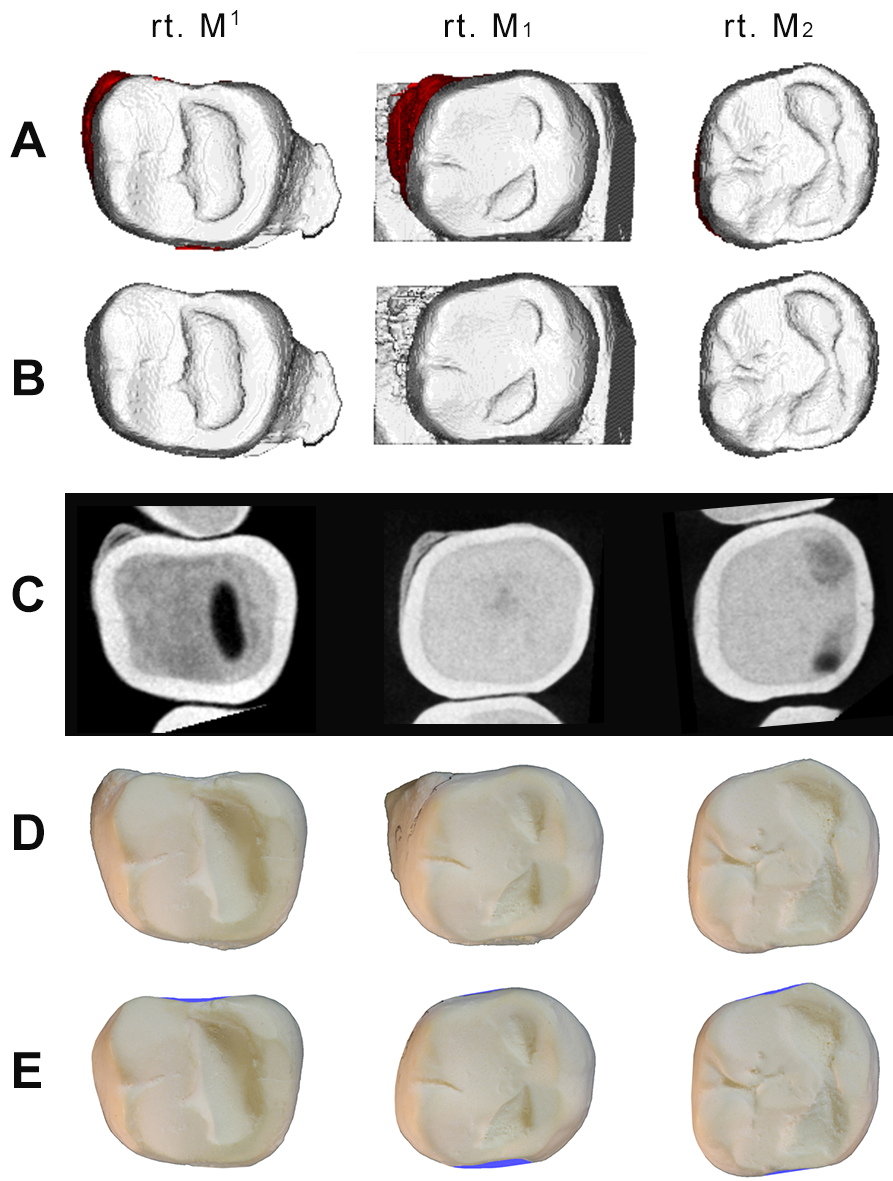

Supplement: S3 Fig — Calculus was identified (red) (A), and removed virtually (B) in the micro-CT imagery. Calculus was clearly distinguishable from the enamel as parts with lesser CT values, as seen in horizontal CT sections (C). A digital photographic image of the high-quality plaster cast was prepared for each tooth, and its background was removed semi-automatically using an image-processing software (D). Then, the calculus was deleted by superimposing the image of B onto D, and worn parts of the tooth were reconstructed with reference to the 3D topography of the plaster cast (blue) to prepare the final image for contour extraction (E). (TIF) [file pone.0141614.s003.tif]
