# Supplementary material for: Unique Dental Morphology of Homo floresiensis and Its Evolutionary Implications
Source: PLoS One. 2015 Nov 18;10(11):e0141614. doi: 10.1371/journal.pone.0141614 (PMC4651360; doi:10.1371/journal.pone.0141614)
Supplement: S1 Table — (PDF) [file pone.0141614.s004.pdf]

S1 Table. Comparative Early Pleistocene *Homo* samples[a].

Sample	Specimen	Data source and references
H. habilis s. l. (2.3-1.6 Ma *Homo* from East Africa)		
Hadar	A.L. 666-1	[1]
Omo (Member G/H)	L7-279*, 628-224, 894-1*; Omo 29-43*, 74-18*, 75-14*,75s-15*, 75s-16, 75i-1255, 195-1630*, SH1-1-17*	casts, [2,3]
East Turkana (upper Burgi and lower KBS)	KNM-ER 1462, 1470, 1480, 1483, 1501, 1502*, 1507*, 1508*, 1590*, 1801*, 1802*, 1805*, 1811, 1812, 1813*, 2597*, 2599, 2601*, 3734, 3891, 3953, 60000, 62000, 62003	casts/originals, [3,4]
Olduvai (Bed I/lower Bed II)	OH 4, 6*, 7*, 13*, 15, 16*, 21*, 24*, 27, 37*, 39*, 41*, 42, 44*, 45*, 55*, 57, 58, 60, 62, 65, 69, 73	casts/originals, [3,5,6]
H. ergaster (1.7-0.8 Ma *Homo* from East Africa)		
East Turkana (upper KBS and Okote)	KNM-ER 730, 803, 806*, 807, 808*, 819, 820*, 992*, 1506*, 1515, 1808*, 1814*, 3733*	casts/originals, [3]
West Turkana (1.47 Ma)	KNM-WT 15000	[7]
Omo (Member K)	Omo K7-1*	cast, [2]
Konso	KGA 10-1*, 11-350*	casts, [8]
Olduvai (Bed III/IV)	OH 22*, 51*	casts/originals
**Dmanisi *Homo*** (1.75 Ma)		
Dmanisi	D2600, 2282/211, 2700/2735	[9,10]
**early Javanese *H. erectus*** (>1.2–0.8 Ma)		
Sangiran Lower (Grenzbank or below)	S1b*, 4*, 5*, 6a*, 6b*, 7* (N=27), 8, 9*, 15a, 22*, 27*; Bk7905*; Bpg 2001.04	casts/originals, [11,12]
Sangiran Upper (above Grenzbank)	S7* (N=18), 17*, 21, Skull IX*; Sb 8103*, Ng 8503*, Bu 9706; Njg 2005.05	cast/original, [13-16]
Sangiran (stratigraphy unknown)	Ng 9603*	cast/original
**East Asian Middle Pleistocene archaic *Homo***		
Zhoukoudian Locality 1	Sin 1–117, 130'–147'; Zdansky premolar & molar; PA 67–70, 110	casts/originals, [17,18]
Lantian, Hexian, Changyang, Chaoxian, Xujiayao, Penghu[b]	PA 102 (Lantian); PA 832–839, HXUP3 (Hexian); V. 1681, 1682 (Changyang); Chaoxian; PA 1480, 1481, 1496, 1500 (Xujiayao); Penghu 1	originals, [18-23]

*Samples included in the EFAs.

[a]The original fossil specimens listed above are housed at the National Museum of Ethiopia, Addis Ababa (Hadar, Omo, Konso), National Museum of Kenya, Nairobi (Turkana), National Museum and House of Culture, Dar es Salaam, and National Natural History Museum, Arusha (Olduvai), Georgian National Museum, Tbilisi (Dmanisi), Senckenberg Research Institute, Frankfurt-am-Main, Geological Museum, Bandung, Bandung Institute of Technology, Bandung, and Gadjah Mada University, Yogyakarta (Sangiran), Institute of Vertebrate Paleontology and Paleoanthropology, Beijing (Zhoukoudian, Lantian, Hexian, Changyang, Chaoxian, Xujiayao), and National Museum of Natural Science, Taichung (Penghu).

[b]Specimen possibly belonging to the Late Pleistocene [23].

References

1. Kimbel WH, Johanson DC, Rak Y. Systematic assessment of a maxilla of *Homo* from Hadar, Ethiopia. Am J Phys Anthropol. 1997; 103: 235-262.

2. Suwa G. A comparative analysis of hominid dental remains from the Sungura and Usno Formations, Omo Valley, Ethiopia. PhD dissertation, University of California at Berkeley; 1990.

3. Wood B. Koobi Fora Research Project 4: Hominid Cranial Remains. Oxford: Clarendon Press; 1991.

4. Leakey MG, Spoor F, Dean MC, Feibel CS, Anton SC, et al. New fossils from Koobi Fora in northern Kenya confirm taxonomic diversity in early *Homo*. Nature. 2012; 488: 201-204.

5. Clarke RJ. A *Homo habilis* maxilla and other newly-discovered hominid fossils from Olduvai Gorge, Tanzania. J Hum Evol. 2012; 63: 418-428.

6. Tobias PV. Olduvai Gorge, 4: the skulls, endocasts and teeth of *Homo habilis*. Cambridge: Cambridge University Press; 1991.

7. Brown B, Walker A. The dentition. In: Walker A, Leakey R, editors. The Nariokotome *Homo erectus* skeleton. Cambridge, MA: Harvard Univesity Press; 1993. pp. 161-192.

8. Suwa G, Asfaw B, Haile-Selassie Y, White T, Katoh S, et al. Early Pleistocene *Homo erectus* fossils from Konso, southern Ethiopia. Anthropol Sci. 2007; 115: 133-151.

9. Martinón-Torres M, Bermúdez de Castro JM, Gómez-Robles A, Margvelashvili A, Prado L, et al. Dental remains from Dmanisi (Republic of Georgia): morphological analysis and comparative study. J Hum Evol. 2008; 55: 249-273.

10. Lordkipanidze D, Ponce de Leon M, Margvelashvili A, Rak Y, Rightmire GP, et al. A complete skull from Dmanisi, Georgia, and the evolutionary biology of early *Homo*. Science. 2013; 342: 326-331.

11. Kaifu Y, Baba H, Aziz F, Indriati E, Schrenk F, et al. Taxonomic affinities and evolutionary history of the Early Pleistocene hominids of Java: dentognathic evidence. Am J Phys Anthropol. 2005; 128: 709-726.

12. Zaim Y, Ciochon RL, Polanski JM, Grine FE, Bettis EA, 3rd, et al. New 1.5 million-year-old *Homo erectus* maxilla from Sangiran (Central Java, Indonesia). J Hum Evol. 2011; 61: 363-376.

13. Baba H, Aziz F, Narasaki S, Sudijono, Kaifu Y, et al. A new hominid incisor from Sangiran, Central Java. J Hum Evol. 2000; 38: 855-862.

14. Kaifu Y, Aziz F, Baba H. Hominid mandibular remains from Sangiran: 1952-1986 collection. Am J Phys Anthropol. 2005; 128: 497-519.

15. Kaifu Y. Advanced dental reduction in Javanese *Homo erectus*. Anthropol Sci. 2006; 114: 35-43.

16. Kaifu Y, Arif J, Yokoyama K, Baba H, Suparka E, et al. A new *Homo erectus* molar from Sangiran. J Hum Evol. 2007; 52: 222-226.

17. Weidenreich F. The dentition of Sinanthropus pekinensis: a comparative odontography of the hominids. Palaeontol Sin New Ser D. 1937; 1: 1-180.

18. Liu W, Wu X, Xing S, Zhang Y. Human Fossils in China. Beijing: Science Press; 2014.

19. Wu X, Poirier FE. Human Evolution in China Oxford: Oxford University Press; 1995.

20. Bailey SE, Liu W. A comparative dental metrical and morphological analysis of a Middle Pleistocene hominin maxilla from Chaoxian (Chaohu), China. Quat Int. 2010; 211: 14-23.

21. Xing S, Martinón-Torres M, Bermúdez de Castro JM, Zhang Y, Fan X, et al. Middle Pleistocene hominin teeth from Longtan Cave, Hexian, China. PLoS ONE. 2014; 9(12): e114265.

22. Xing S, Martinón-Torres M, Bermúdez de Castro JM, Wu X, Liu W. Hominin teeth from the early Late Pleistocene site of Xujiayao, Northern China. Am J Phys Anthropol. 2015; 56: 224-240.

23. Chang CH, Kaifu Y, Takai M, Kono RT, Grun R, et al. The first archaic *Homo* from Taiwan. Nat Commun. 2015; 6: 6037.
