# Supplementary material for: Unique Dental Morphology of Homo floresiensis and Its Evolutionary Implications
Source: PLoS One. 2015 Nov 18;10(11):e0141614. doi: 10.1371/journal.pone.0141614 (PMC4651360; doi:10.1371/journal.pone.0141614)
Supplement: S2 Table — (PDF) [file pone.0141614.s005.pdf]

S2 Table. Comparative *Homo sapiens* sample.

	Remarks	N[a]	Repository[b]
**Prehistoric Southeast Asia**			
Flores*	Aimere, Gua Alo, Gua Nempong, Liang Bua, Liang Momer, Liang Toge, Liang X	9	NBC, ARKENAS
Java*	Hoekgrot, Wajak	3	NBC
Malaysia*	Guar Kepah	19	NBC
Vietnam*	Mai Da Dieu, Mai Da Nuoc, Hang Chim, Dong Cang, Con Co Ngua	73	IAH
**Australia/Melanesia**			
New Guinea*		30	AMNH, MH
Australia/Tasmania Aborigine*		19	AMNH
**Southeast Asia**			
Philippine Negrito*		20	MH
Others	Andaman, Indonesia, Malaysia, Nicobar, Philippine, Singapole, Thailand	57	AMNH, MH
**Northeast Asia**			
Northeast Asia	China, Chukuci, Korea, Mongol, Yukagir	18	AMNH
**Africa**			
Bushman		17	AMNH, MH
African Pygmy*		20	MH
South Africa	Excluding Bushman	26	AMNH
East Africa		45	AMNH
West Africa	Excluding Pygmy	55	AMNH
**Indo/Europe**			
India		6	AMNH
German		65	AMNH
Others	Hungary, Poland, Sweden	8	AMNH
Total		490	

*Samples included in the EFAs.

[a]Number of individuals.

[b]NBC = Naturalis Biodiversity Center, Leiden; ARKENAS = National Research and Development Centre for Archaeology, Jakarta; AMNH = American Museum of Natural History, NY; MH = Musee de l'Homme, Paris, IAH = Institute of Archaeology, Hanoi.
