# Supplementary material for: Unique Dental Morphology of Homo floresiensis and Its Evolutionary Implications
Source: PLoS One. 2015 Nov 18;10(11):e0141614. doi: 10.1371/journal.pone.0141614 (PMC4651360; doi:10.1371/journal.pone.0141614)
Supplement: S3 Table — (PDF) [file pone.0141614.s006.pdf]

S3 Table. Results of the non-metric and linear metric comparisons.

		H. habilis	*H. erg.*	Dmanisi	eJ *H. erectus* (Lower, Upper)[c]	MP East Asia	*H. sapiens*	*H. floresiensis* (status)[d]
**Non-metric comparisons of individual teeth** (frequency and ratio)[a]								
1	C_1 distal shoulder low	1/1	−	−	2/2**	1/1	*3/109	1/1 (EP-MP)
	(vs. high)						3%	
2	P^3 transverse crest present	5/11**	1/5	0/1	3/9** (2/5, 1/4)	1/8	**6/283	1/1 (EP)
		45%	20%		33%	13%	2%	
3	P^4 transverse crest present	3/10**	2/3**	1/2*	4/8** (1/4, 3/4)	1/8	**5/279	1/1 (EP)
		30%			50%	13%	2%	
4	P^3 buccal groove(s) present	12/12**	**1/5	0/1	6/9 (4/5, 2/4)	**2/6	**139/245	0/1 (post-Hh)
		100%	20%		67%	33%	57%	
5	P^4 buccal groove(s) present	6/10	3/3*	0/2	1/8 (0/4, 1/4)	3/6	67/217	0/1 (?)
		60%			12%	50%	31%	
6	P^4 lingual crown MD extensive	8/8**	1/2	2/2*	4/4** (2/2, 2/2)	*2/6	**33/198	1/1 (EP)
		100%			100%	33%	17%	
7	P_3 lingual cusp posi. mesially	9/9	*3/6	1/2	3/3 (2/2, 1/1)	8/9	163/197	0/3 (post-Hh)
	(vs. distally)	100%	50%			89%	83%	
8	P_4 lingual cusp posi. mesially	8/8	4/5	2/2	6/6 (4/4, 2/2)	4/5	155/211	1/1 (?)
	(vs. centrally or distally)	100%	80%		100%	80%	73%	
9	P_3 mesiolingual crown	0/9	0/6	0/2	0/4 (0/3, 0/1)	0/9	0/214	3/3 (unique)
	beveled and wrinkled	0%	0%		0%	0%	0%	
10	P_3 transverse crest present	9/9	5/5	2/2	4/4 (3/3, 1/1)	7/8	176/215	3/3 (?)
		100%	100%		100%	88%	82%	
11	P_4 transverse crest present	0/7	*5/7**	0/2	*4/6* (3/4, 1/2)	**5/6**	40/220	2/2 (post-Hh)
		0%	71%		67%	83%	18%	
12	P_3 buccal groove(s) present	7/8**	3/4	0/2	2/3 (2/2, 0/1)	6/9	**57/170	0/3 (?)
		87%	75%			67%	34%	
13	P_4 buccal groove(s) present	4/6	4/5*	0/2	4/5* (2/3, 2/2)	6/7**	51/164	0/1 (?)
		67%	80%		80%	86%	31%	
14	P_3 buccal basal enamel	1/6*	0/5	1/3?*	1/3* (1/3, −)	1/7*	*0/207	1/3 (EP-MP)
	thickened	17%	0%			14%	0%	
15	P_3 root bifurcated	4/13**	2/5*	1/1	3/6** (3/6, −)	0/7	**26/599[e]	2/3 (EP)
	(vs. fused or single)	31%	40%		50%	0%	4%	
16	P_4 root bifurcated	5/10**	4/7**	−	4/7** (4/6, 0/1)	0/4	**15/570[e]	0/3 (?)
	(vs. fused or single)	50%	57%		57%	0%	3%	
17	M_1 four-cusped	0/13	0/10	0/2	0/9 (0/7, 0/2)	0/14	9/268	2/2 (Hs)
	(vs. five-cusped)	0%	0%		0%	0%	3%	
18	M_2 four-cusped	0/9**	0/8**	0/2	0/15** (0/12, 0/3[f])	0/12**	**163/279	2/2 (Hs)
	(vs. five-cusped)	0%	0%		0%	0%	58%	
19	M_1 mid-trigonid crest present	0/7	1/9	2/2	4/9** (3/6, 1/3)	1/9	7/176	1/1
		0%	11%		44%	11%	4%	
20	M_2 mid-trigonid crest present	1/7	2/7	1/2	1/13 (1/10, 0/3)	0/11	6/265	1/1
		14%	29%		8%	0%	2%	
**Metric comparisons of dentition as a whole** (minimum / maximum, and sample size in the lower rows)[b]								
21	Relative P_3 size (%)	24 / 26	26 / 28	26 / 27	(26, −)	26 / 28	22 / 27	30 (unique)
		N = 5	N = 3	N = 2	N = 1, −	N = 3	N = 188	N = 2
22	Molar size % increase: $M_1 \rightarrow M_2$	+5 / +20	−2 / +11	−2 / +8	(+4 / +9, +4 / +7)	−2 / +4	−15 / +5	0 / +1 (post-Hh)
		N = 7	N = 6	N = 3	N = 3, 2	N = 6	N = 250	N = 2
	Molar size % increase: $M_2 \rightarrow M_3$	−9 / +7	−10 / −2	−8 / +8	(−10 / 0, −9)	−14 / 0	−15 / +14	−9 / −6 (?)
		N = 6	N = 3	N = 2	N = 4, 1	N = 5	N = 211	N = 2
23	Alv. arcade index (Lth/Bth, %)	117 / 156	98	123	(107 / 108, −)	88	−	103 / 105 (eJHe)
		N = 5	N = 1	N = 2	N = 2, −	N = 1	−	N = 2

[a]Observed frequencies and percent ratios (for those samples with N ≥ 4) as well as the results of the Fisher's exact tests for the non-metric comparisons (nos. 1−20). The asterisk(s) to the right of each frequency indicates significant difference from the *H. sapiens* sample, and that on the left significant differences from the *H. habilis* sample (*: P < 0.05, **: P < 0.01).

[b]The minimum and maximum values, and sample sizes (in the lower rows) are shown for the metric comparisons (nos. 21−23).

[c]Frequencies for the Sangiran Lower and Upper subsamples in the parentheses.

[d]Morphological status of *H. floresiensis* in the parentheses: 'EP', a primitive condition shared with the Afro-Asian Early Pleistocene *Homo*; 'EP-MP', a primitive condition shared with the Afro-Asian Early Pleistocene *Homo* and the East Asian Middle-Late Pleistocene archaic *Homo*; 'post-Hh', a condition derived from *H. habilis*; 'Hs', a derived condition shared with *H. sapiens*; 'eJHe', a condition most similar to early Javanese *H. erectus*; 'unique', a unique condition restricted to *H. floresiensis*.

[e]Sample studied by Shields [1].

[f]Zanolli [2] recently suggested the presence of 4-cusped M_2s in this group.

References

1. Shields ED. Mandibular premolar and second molar root morphological variation in modern humans: What root number can tell us about tooth morphogenesis. Am J Phys Anthropol. 2005; 128: 299-311.

2. Zanolli C. Additional evidence for morpho-dimensional tooth crown variation in a New Indonesian *H. erectus* sample from the Sangiran Dome (Central Java). PLoS ONE. 2013; 8: e67233.
