# Supplementary material for: Unique Dental Morphology of Homo floresiensis and Its Evolutionary Implications
Source: PLoS One. 2015 Nov 18;10(11):e0141614. doi: 10.1371/journal.pone.0141614 (PMC4651360; doi:10.1371/journal.pone.0141614)
Supplement: S1 Text — (PDF) [file pone.0141614.s008.pdf]

S1 Text. Additional notes on the non-metric and linear metric comparisons

This section reports the results and notes for the non-metric and linear metric comparisons that were not included in the main text. These were found not to be useful to evaluate dental morphological status of *H. floresiensis*.

P^4 buccal grooves (no. 5 in S3 Table). This trait was assessed as was done for P^3 buccal grooves (no. 4 in Table 2). The frequencies of appearance of the grooves in P^4 decrease from *H. habilis* (60%) to later *Homo* (30% in the pooled post-*habilis* Early Pleistocene sample and 31 % in the *H. sapiens*), although the difference was statistically significant only between the *H. ergaster* and the *H. sapiens* samples.

P_4 lingual cusp position (no. 8 in S3 Table)**.** We recorded this character using similar to but slightly different from the criterion applied for P_3. Here, the lingual cusp (metaconid) positioned opposite to the buccal cusp (protoconid) was recorded as distal, not mesial as was the case for P_3. In the Early Pleistocene *Homo*, a distally positioned lingual cusp is rare (1/21 in our sample: Table 2). Although *H. sapiens* shows a slightly higher frequency of this cusp arrangement, the mesially positioned lingual cusp arrangement is still the dominant condition in our species (73%).The single *H. floresiensis* P_4 available to assess this trait (LB6/1: Fig 1G, 1H) has a mesially positioned lingual cusp.

P_3 transverse crest (no. 10 in S3 Table)**.** This trait was recorded as was done for P_4 transverse crest (no. 11 in Table 2). All the Early Pleistocene P_3s we investigated have a transverse crest (N = 20), but this structure is absent in some (12–18%) of the East Asian Middle Pleistocene archaic *Homo* and *H. sapiens* specimens (S3 Table). Although these small differences are not statistically significant, the lack of this structure is a derived condition because it is commonly present in earlier hominins including *Australopithecus* [1-6] and *Ardipithecus* [7], but occasionally undeveloped in later hominins such as *Paranthropus* (23%) [8,9] and the European Middle Pleistocene *Homo* (21%) [10]. The P_3s from three *H. floresiensis* individuals have well-developed transverse crests (Fig 1A, 1B, 1E, 1G, 1H). The EDJ surface morphology of LB1 is consistent with this assessment (Fig 1A).

P_3 and P_4 buccal grooves (nos. 12 and 13 in S3 Table). Buccal grooves (mesial and distal buccal

grooves) on the mandibular premolars were recorded using the same methods used for the maxillary premolars (nos. 5 and 6). Previous studies showed that African earlier *Homo* is characterized by weaker expression of distal buccal groove than in *Au. afarensis* and *Au. africanus* in the mandibular premolars [8,9,11]. In our *Homo* sample, in both P_3 and P_4, the observed frequency decrease markedly from the Early-Middle Pleistocene archaic *Homo* groups (67–87%) to the *H. sapiens* (31–34%) samples, with significant differences detected between the pooled Early Pleistocene *Homo* and the *H. sapiens* samples (P = 0.004 [P_3] and 0.0009 [P_4], Fisher's exact tests, not indicated in Table 2). Therefore, the absence of buccal grooves in the three P_3s (LB1, LB2/2, LB6/1) and one P_4 (LB6/1) of *H. floresiensis* may be a derived condition. However, the polymorphic nature of this trait in all the samples compared here [8,9] makes such an evaluation inconclusive.

P_4 root form (no. 16 S3 Table)**.** This trait was recorded as was done for P_3 root form (no. 15 in Table 2). The results in Table 2 show that this trait is polymorphic in most samples compared here [12], but the Early Pleistocene *Homo* samples show bifurcated roots significantly more than in the large global modern human sample studied by Shield [13]. Two individuals of *H. floresiensis* (LB1, 6/1) have fused (Tomes') roots and the other individual (LB15/1) has a single but BL broad and robust root [14]. Because fused or single P_4 root is common in both the Early Pleistocene (about 50%) and modern (97%) *Homo*, taxonomic significance of the observed frequency of fused/single P_4 root in *H. floresiensis* (3/3) is difficult to evaluate. Shields [13] found that smaller-toothed modern human populations tend to have simplified premolar root morphology. This suggests that the high frequency of fused/single P_4 root in *H. floresiensis* may be consequence of its small crown size (Fig 2).

M_2 mid-trigonid crest (no. 20 in S3 Table)**.** This trait was recorded as was done for M_1 mid-trigonid crest (no. 19 in Table 2). It is relatively rare in all the *Homo* samples compared here and none of the pair-wise comparisons showed statistically significant differences. The EDJ surface morphology of the LB1 M_2 strongly suggests the crest was originally present on the enamel surfaces of this tooth as was the case for its M_1 (Fig 1C) [14].

Molar size proportion (no. 22 in S3 Table)**.** We compared percent increases of the 'tooth crown size' (square root of the calculated crown area [MD × BL]) from M_1 to M_2 ([M_2−M_1]/M_1), and from M_2 to M_3 ([M_3−M_2]/M_2). The result for the former is described in the main text. The M_2-M_3 % size increase is considerably variable in most samples examined here. Both the earliest (*H. habilis*) and

latest (*H. sapiens*) *Homo* show great variations, with the average conditions close to $M_2 = M_3$ (+1% and −2% for *H. habilis* and *H. sapiens*, respectively). Because the values for *H. floresiensis* (LB1, 6/1) are encompassed within these variations, this trait is not very useful in evaluating the primitive/derived status of the *H. floresiensis* teeth (Fig 5E). There is a weak correlation between this variable and the crown size ($M_2 + M_3$) in the *H. sapiens* sample (r = 0.299).

References

1. Robinson JT. The Dentition of the Australopithecinae. Pretoria: Transvaal Museum; 1956.

2. White TD. New fossil horninids from Laetolil, Tanzania. Am J Phys Anthropol. 1977; 46: 197-230.

3. Johanson DC, White TD, Coppens Y. Dental remains from the Hadar Formation, Ethiopia: 1974-1 977 collections. Am J Phys Anthropol. 1982; 57: 545-603.

4. Ward CV, Leakey MG, Walker A. Morphology of *Australopithecus anamensis* from Kanapoi and Allia Bay, Kenya. J Hum Evol. 2001; 41: 255-368.

5. Ward CV, Manthi FK, Plavcan JM. New fossils of *Australopithecus anamensis* from Kanapoi, West Turkana, Kenya (2003-2008). J Hum Evol. 2013; 65: 501-524.

6. Moggi-Cecchi J, Grine FE, Tobias PV. Early hominid dental remains from Members 4 and 5 of the Sterkfontein Formation (1966-1996 excavations): catalogue, individual associations, morphological descriptions and initial metrical analysis. J Hum Evol. 2006; 50: 239-328.

7. Suwa G, Kono RT, Simpson SW, Asfaw B, Lovejoy CO, et al. Paleobiological implications of the *Ardipithecus ramidus* dentition. Science. 2009; 326: 69-69, 94-99.

8. Suwa G. A comparative analysis of hominid dental remains from the Sungura and Usno Formations, Omo Valley, Ethiopia. PhD dissertation, University of California at Berkeley. 1990

9. Wood B, Uytterschaut H. Analysis of the dental morphology of Plio-Pleistocene hominids. III. Mandibular premolar crowns. J Anat. 1987; 154: 121-156.

10. Martinón-Torres M, Bermúdez de Castro JM, Gómez-Robles A, Prado-Simón L, Arsuaga JL. Morphological description and comparison of the dental remains from Atapuerca-Sima de los Huesos site (Spain). J Hum Evol. 2012; 62: 7-58.

11. Suwa G, White TD, Howell FC. Mandibular postcanine dentition from the Shungura Formation, Ethiopia: crown morphology, taxonomic allocations, and Plio-Pleistocene hominid evolution. Am J Phys Anthropol. 1996; 101: 247-282.

12. Wood BA, Abbott SA, Uytterschaut H. Analysis of the dental morphology of Plio-Pleistocene hominids. IV. Mandibular postcanine root morphology. J Anat. 1988; 156: 107-139.

13. Shields ED. Mandibular premolar and second molar root morphological variation in modern humans: What root number can tell us about tooth morphogenesis. Am J Phys Anthropol. 2005; 128: 299-311.

14. Kaifu Y, Kono RT, Sutikna T, Saptomo EW, Jatmiko, et al. Descriptions of the dental remains of *Homo floresiensis*. Anthropol Sci. 2015; 123: 129-145.
